# The Paralogous Histone Deacetylases Rpd3 and Rpd31 Play Opposing Roles in Regulating the White-Opaque Switch in the Fungal Pathogen *Candida albicans*

**DOI:** 10.1128/mBio.01807-16

**Published:** 2016-11-15

**Authors:** Jing Xie, Sabrina Jenull, Michael Tscherner, Karl Kuchler

**Affiliations:** Department of Medical Biochemistry, Medical University of Vienna, Max F. Perutz Laboratories, Campus Vienna Biocenter, Vienna, Austria

## Abstract

Chromatin modifications affect gene regulation in response to environmental stimuli in numerous biological processes. For example, *N*-acetyl-glucosamine and CO_2_ induce a morphogenetic conversion between white (W) and opaque (O) cells in *MTL* (mating-type locus) homozygous and heterozygous (**a**/α) strains of the human fungal pathogen *Candida albicans*. Here, we identify 8 histone-modifying enzymes playing distinct roles in the regulation of W/O switching in *MTL* homozygous and heterozygous strains. Most strikingly, genetic removal of the paralogous genes *RPD3* and *RPD31*, both of which encode almost identical orthologues of the yeast histone deacetylase (HDAC) Rpd3, reveals opposing roles in W/O switching of *MTL***a**/α strains. We show that Rpd3 and Rpd31 functions depend on *MTL* genotypes. Strikingly, we demonstrate that Rpd3 and Rpd31, which are almost identical except for a divergent C-terminal extension present in Rpd31, exert their functions in distinct regulatory complexes referred to as CaRpd3L and CaRpd31S complexes. Moreover, we identify the *Candida* orf19.7185 product Ume1, the orthologue of yeast Ume1, as a shared core subunit of CaRpd3L and CaRpd31S. Mechanistically, we show that the opposing roles of Rpd3 and Rpd31 require their deacetylase activities. Importantly, CaRpd3L interacts with the heterodimeric transcriptional repressor **a**1/α2, thus controlling expression of *WOR1* encoding the master regulator of W/O switching. Thus, our work provides novel insight about regulation mechanisms of W/O switching in *MTL***a**/α strains. This is the first example of two highly conserved HDACs exerting opposing regulatory functions in the same process in a eukaryotic cell.

## IMPORTANCE

*RPD3*-like histone deacetylases (also called class I HDACs) are conserved from unicellular eukaryotes to mammals. Specifically, the genome of the human fungal pathogen *Candida albicans*, the most frequent cause of invasive fungal infections of high morbidity and mortality, harbors two almost identical paralogous HDACs, Rpd3 and Rpd31. We show here for the first time that Rpd3 and Rpd31 acquired functional divergence related to a distinct C-terminal domain. Rpd3 and Rpd31 associate with different complexes in the control regions of the master regulator gene *WOR1*, which is required for white-opaque (W/O) morphogenesis, respectively. The ability to switch is important for fungal pathogenesis, since it enables distinct host niche colonization. This work is to the best of our knowledge the first description of two paralogous HDACs playing opposing functional roles in the same developmental process. Our work adds a new angle concerning the molecular understanding of HDACs in the regulation of cell fate decisions.

## INTRODUCTION

In eukaryotic cells, posttranslational modifications (PTMs) of histones dynamically modulate chromatin architecture and function, which play pivotal roles in numerous biological processes in response to environmental stimuli (1, 2). In fungal pathogens, adaptive chromatin changes promote phenotypic plasticity implicated in many development processes. For example, chromatin remodeling in human fungal pathogens such as *Candida* spp. enables their rapid adaptation to host niches and allows for colonization or infection (3). *Candida albicans*, the most common human opportunistic fungal pathogen (4), can cause diseases ranging from superficial to life-threatening systemic infections (5). The ability to mount morphogenetic changes such as filamentation in response to different host stimuli is a key virulence trait of *C. albicans* (6, 7). Moreover, in addition to filamentation (6), *C. albicans* can undergo a reversible switch between two morphologies known as white (W) and opaque (O) phases (8). W cells are oval and form dome-shaped colonies, while O cells are elongated and form flatter and darker colonies, which can be selectively stained on plates containing the dye phloxine B (9). Although the W and O cell types share the same genome, they display distinct phenotypes, including filamentation, adhesion to human tissues and plastics, proteinase production, and mating competence, as well as antifungal drug susceptibility (10, 11). More importantly, O cells acquire hypervirulence phenotypes, at least in cutaneous infections, and appear less well recognized by the host immune surveillance (12, 13).

For decades, white-opaque (W/O) switching was presumed to occur exclusively in *MTL* homozygous mating-type (**a**/**a**, α/α) strains of *C. albicans* (11). The heterodimeric **a**1/α2 transcriptional regulator acts in heterozygous *MTL***a**/α strains to repress the expression of Wor1, the key regulator of W/O switching. Thus, **a**1/α2 blocks cells in the W state (14). However, recent work discovered O cell formation in a number of natural *MTL***a**/α strains in response to environmental cues such as *N*-acetylglucosamine (GlcNAc) or CO_2_, suggesting that phenotypic switching competency is a common trait shared by all *MTL* types of *C. albicans* (12). Of note, regulation of W/O switching involves a dual layer of both transcriptional and epigenetic networks (15). At least seven transcriptional regulators, Wor1, Wor2, Wor3, Wor4, Czf1, Efg1, and Ahr1, form a network of positive and negative feedback loops to control W/O conversion (16–18). Furthermore, at the epigenetic level, several histone-modifying enzymes strongly impact W/O switching frequencies (15, 19, 20). For example, the SET3C histone deacetylase (HDAC) complex operates in concert with transcription factors to modulate W/O switching (15), as well as filamentation (21) and biofilm formation (22). However, the impact of HDACs on the modulation of W/O switching in *MTL***a**/α cells has not been extensively investigated, although at least 5 transcription factors, Wor1, Rfg1, Brg1, Efg1, and Wor4, have been implicated to date (12, 18).

Environmental stimuli may help **a**/α cells to override the inhibition of *WOR1* by the **a**1/α2 repressor complex and thus facilitate W/O switching. Of note, emerging evidence suggests the epigenetic integration of extracellular and intrinsic signaling events (23, 24). Here, we use a systematic reverse-genetics approach to show that 8 out of 20 deletion mutants lacking histone-modifying enzymes play different roles in the regulation of W/O switching in *MTL* homozygous versus heterozygous strains. Most remarkably, genetic ablation of *RPD3* and *RPD31*, two paralogous genes encoding almost identical HDACs, shows that these genes play opposing roles in W/O switching in *MTL***a**/α strains. Moreover, we demonstrate for the first time that Rpd3 and Rpd31 form distinct regulatory complexes recruiting distinct proteins, most likely due to a short divergent C-terminal tail present in Rpd31. Rpd3-mediated regulation of W/O switching requires the master regulator gene *WOR1* (25). Interestingly, the regulatory role of Rpd3 in *MTL* homozygous versus heterozygous strains requires HDAC activity, which modulates recruitment of the heterodimeric **a**1/α2 repressor to the *WOR1* promoter. We propose a model whereby Rpd3 and Rpd31 control W/O switching in *MTL***a**/α strains. Thus, in addition to novel insights into the epigenetic regulation of developmental switches, our data exemplify how paralogous genes in a pathogen can maintain the same enzymatic activity but undergo functional diversification following gene duplication events. In the case of *C. albicans*, we propose that this diversification allows for flexible adaption to changing host niches and promotes or sustains host colonization.

## RESULTS

### Deletion of histone-modifying genes affects W/O switching in *MTL*a/α strains.

**FIG 1:**
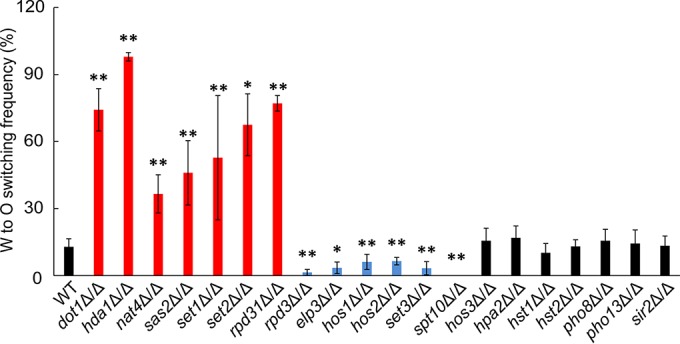
Genetic removal of histone modifier genes affects W/O switching. All homozygous deletion mutants were constructed in the *MTL* heterozygous recipient wild-type strain J4-2.1 (WT *MTL***a**/α). Strains were grown on YPD plates at 30°C for 2 days. Then W cells were plated on Lee’s GlcNAc medium at 25°C with 5% CO_2_ for 5 days to quantify W/O switching. Frequencies were calculated as follows: [(opaque + opaque-sectored colonies)/total colonies] × 100. *, *P* < 0.05, and **, *P* < 0.01, compared to the WT strain.

Previous studies have shown that host environmental stimuli can override the inhibition by the **a**1/α2 complex and induce W/O switching in *MTL***a**/α strains (12). However, the underlying mechanisms remain unclear. Since chromatin modifications play important roles in response to environmental stimuli, we systematically tested the roles of histone modifiers in modulating W/O switching in *MTL***a**/α strains. In total, we constructed 20 homozygous deletion mutants lacking histone-modifying enzymes involved in histone acetylation, deacetylation, methylation, and dephosphorylation in an *MTL***a**/α background. W/O switching assays under optimal inducing conditions (12) showed that 13 out of 20 genes altered W/O switching frequencies compared to the wild-type (WT) control (Fig. 1).

Five mutants (*hda1*Δ/Δ, *rpd31*Δ/Δ, *set1*Δ/Δ, *hos2*Δ/Δ, and *set3*Δ/Δ) showed similar effects in *MTL* homozygous and heterozygous strains (15, 20). However, *dot1*Δ/Δ strains lacking an H3K79 methylase (26), the *sas2*Δ/Δ mutant devoid of a histone acetyltransferase (HAT) (27), and the *set2*Δ/Δ methyltransferase mutant (28) showed increased O cell formation. Conversely, *elp3*Δ/Δ mutants lacking a putative HAT (29) and the *hos1*Δ/Δ mutant lacking an HDAC (20) displayed significantly decreased W/O switching. Of note, deletion of *SPT10*, encoding a putative histone H3 acetyltransferase (30), completely blocked O cell formation in *MTL***a**/α strains (Fig. 1). Notably, none of these six genes influenced switching in *MTL* homozygous cells (15). Removal of the *NAT4* HAT increased W/O switching, while it showed decreased conversion in *MTL* homozygous strains (15). Most surprisingly, deletion of *RPD3* strongly decreased W/O switching in *MTL***a**/α strains, whereas the paralogous gene *RPD31* displayed an opposing function (Fig. 1). Since the putative HADCs Rpd3 and Rpd31 share a high primary sequence identity of 74% (see Fig. S1 in the supplemental material), we wondered how almost identical HDACs can play inverse functions in W/O switching in *C. albicans*.

### Opposing effects of *RPD3* and *RPD31* on W/O switching are MTL dependent.

**FIG 2:**
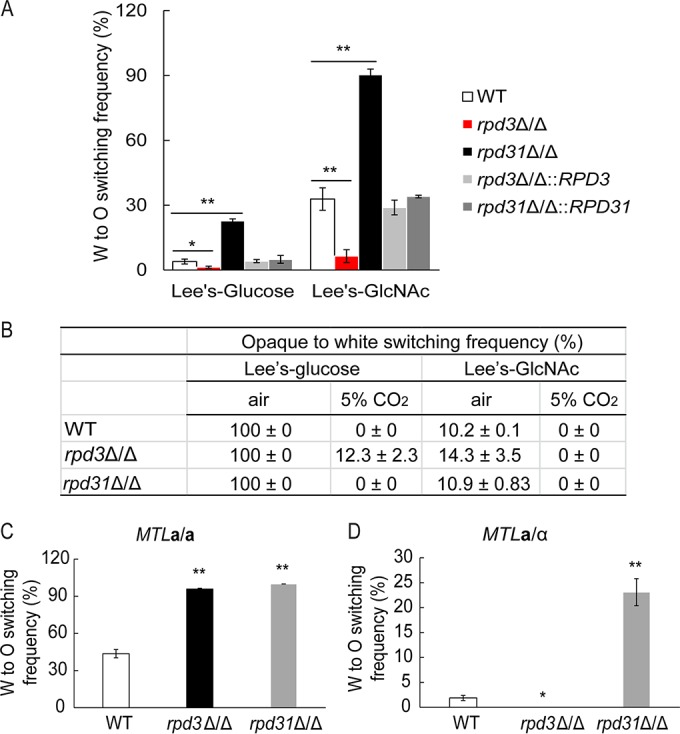
*RPD3* and *RPD31* play opposing roles in W/O switching of *MTL* heterozygotes. (A) Genetic deletion of *RPD3* and *RPD31* in *MTL***a**/α cells showed consistent W/O switching frequencies under different culture conditions. The indicated strains were grown on YPD plates at 30°C for 2 days. Cells were plated on Lee’s glucose medium and Lee’s GlcNAc medium, respectively, and incubated at 25°C with 5% CO_2_ for 5 days. (B) O/W switching of the WT (J4-2.1) and *rpd3*Δ/Δ and *rpd31*Δ/Δ mutant *MTL***a**/α strains under different growth conditions. Pure opaque cells were plated on Lee’s glucose medium and Lee’s GlcNAc medium and cultured under normal aeration or at 25°C with 5% CO_2_ for 5 days, respectively. (C and D) Comparison of W/O switching frequencies of WT, *rpd3*Δ/Δ, and *rpd31*Δ/Δ cells in *MTL***a**/**a** (J130) and *MTL***a**/α (J4-2.1) background genotypes. All strains were grown on YPD plates at 30°C for 2 days. Then cells were plated on SC-glucose medium at 25°C with 5% CO_2_. After 5 days, cells from white colonies were replated on fresh SC-glucose medium at 25°C with 5% CO_2_ for 5 days. *, *P* < 0.05, and **, *P* < 0.01, compared to the WT strain.

The W/O switching assays used media containing GlcNAc as the carbon source, while glucose was used in previous studies (15, 20). Hence, the impact of different carbon sources on W/O switching was tested in different culture media (Fig. 2A). Both *rpd3*Δ/Δ and *rpd31*Δ/Δ mutants showed consistent results in Lee’s glucose medium and Lee’s GlcNAc medium. While lack of Rpd3 strongly decreased switching, loss of Rpd31 dramatically increased the W/O conversion, showing that the opposing roles of Rpd3 and Rpd31 are medium independent (Fig. 2A). Importantly, genomic reintegration of *RPD3* and *RPD31* into the corresponding homozygous deletion mutants fully restored wild-type phenotypes (Fig. 2A). To exclude the possibility that the *rpd3*Δ/Δ and *rpd31*Δ/Δ mutant phenotypes are strain specific, we deleted both genes in another clinical *MTL***a**/α strain from an entirely different source (12). Importantly, we confirmed the results for both genes, showing that lack of *RPD3* decreased W/O switching, whereas lack of *RPD31* increased the conversion (see Fig. S2 in the supplemental material). We also quantified the reverse switching frequency, namely, the O/W conversion of the WT and *rpd3*Δ/Δ and *rpd31*Δ/Δ mutants under four different culture conditions. Only *rpd3*Δ/Δ mutants displayed insignificant increases in the O/W switching on Lee’s glucose medium plus 5% CO_2_ (Fig. 2B), suggesting that both Rpd3 and Rpd31 exert their regulatory roles in W rather than in O cells.

In a previous study, the genetic removal of *RPD3* in *MTL*α/α strains increased W/O switching, but the parent strain used in our study differs from the one used in the previous study (20). Thus, to confirm that the regulation by Rpd3 is indeed different between *MTL* homozygous strains and heterozygous strains, we removed the *RPD3* and *RPD31* genes in an *MTL***a**/**a** cells obtained through a spontaneous conversion from our wild-type *MTL***a**/α parent strain (J4-2.1). However, the WT *MTL***a**/**a** control strain, as well as both *rpd3*Δ/Δ and *rpd31*Δ/Δ mutants, showed 100% W/O switching both on Lee’s glucose medium and Lee’s GlcNAc medium when incubated in 5% CO_2_. Because the strong inducing impact of CO_2_ may mask intrinsic *rpd3*Δ/Δ and *rpd31*Δ/Δ phenotypes, we performed W/O switching assays on synthetic complete (SC)-glucose medium with 5% CO_2_. The result showed that *rpd31*Δ/Δ deletions in both *MTL***a**/**a** and **a**/α strains increased W/O switching frequencies compared to those of the isogenic WT strains (Fig. 2C and D). In sharp contrast, only lack of *RPD3* in *MTL***a**/**a** cells increased W/O switching (Fig. 2C), which is consistent with earlier data (20), while the *rpd3*Δ/Δ mutation in an *MTL***a**/α strain decreased switching (Fig. 2D). Additionally, we also deleted the *MTL***a***1* gene in **a**/α backgrounds of WT, *rpd3*Δ/Δ, and *rpd31*Δ/Δ **a**/α cells. Switching assays performed with these strains yielded similar results as for the *MTL***a**/**a** background (data not shown). Thus, our data demonstrate that *RPD3* regulates W/O switching differently in *MTL* homozygous strains versus heterozygous strains (Fig. 2C).

### Rpd3 and Rpd31 act in distinct regulatory complexes to control W/O switching.

**FIG 3:**
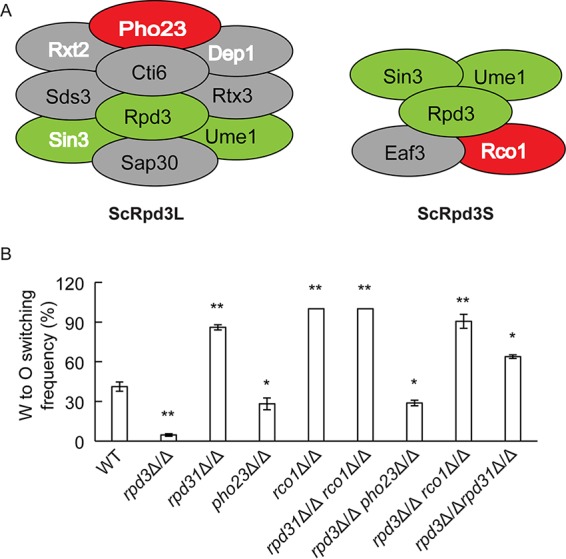
Lack of *RPD3* and *PHO23* debilitates W/O switching, whereas deletion of *RPD31* and *RCO1* promotes switching of *MTL***a**/α cells. (A) Schematic cartoons of Rpd3L and Rpd3S complexes from *S. cerevisiae* (31). Green ovals indicate three core components shared by both complexes; gray and red ovals indicate the components specific to each complex, wherein ovals with white and bold letters indicate homologous components identified in *C. albicans* via immunoprecipitation in the present work. (B) Single and double deletion mutants were constructed in the WT strain (J4-2.1) and confirmed by PCR amplification using genomic DNA directly prepared from colonies. Strains were grown on YPD plates at 30°C for 2 days and then plated on Lee’s GlcNAc medium and grown at 25°C with 5% CO_2_ for 5 days. *, *P* < 0.05, and **, *P* < 0.01, compared to the WT strain.

In *Saccharomyces cerevisiae*, Rpd3 constitutes the catalytic subunit of two well-known HDAC complexes, ScRpd3L and ScRpd3S, and of the newly characterized complex ScRpd3μ (31). In *C. albicans*, Rpd3 and Rpd31 are two putative orthologues of ScRpd3 sharing sequence identities of 75% and 84%, respectively (Fig. S1). Based on the Candida Genome Database, except for Ume1, all other subunits of the ScRpd3L and ScRpd3S complexes are conserved in *C. albicans* (Fig. 3A; see Table S4 in the supplemental material). For example, *ScPHO23* encodes an Rpd3L-specific subunit, whose deletion did not affect Rpd3L complex integrity but significantly decreased the HDAC activity (32). Furthermore, *ScRCO1* encodes an Rpd3S-specific subunit required for the integrity or stability of the Rpd3S complex (33). Since we hypothesized that *C. albicans* Rpd3 and Rpd31 may operate in similar complexes, we deleted the orthologues of *ScPHO23* and *ScRCO1* in *C. albicans* **a**/α strains and tested their roles in W/O switching.

Interestingly, genetic removal of *PHO23* decreased the W/O switching frequency compared to that of the WT strain, thus phenocopying *rpd3*Δ/Δ traits. In contrast, both *rco1*Δ/Δ and *rpd31*Δ/Δ mutants displayed increased switching frequencies. Notably, lack of *PHO23* increased opaque formation in *MTL***a**/**a** cells (17). Hence, the similar phenotypes displayed by Rpd3 and Pho23 in W/O conversion suggest that both proteins function in a complex. Thus, we constructed a series of double mutants to identify possible genetic interactions. The *rpd3*Δ/Δ *pho23*Δ/Δ double mutant showed the same phenotype as the corresponding single mutants. Furthermore, assaying the *rpd31*Δ/Δ *rco1*Δ/Δ strain yielded similar results (Fig. 3B). These data provide compelling evidence that Rpd3 and Pho23 act in the same complex or pathway and cooperate in the W/O switching control, while Rpd31 and Rco1 work together in a distinct complex to inhibit O cell formation. The *rpd3*Δ/Δ *rpd31*Δ/Δ strain and the *rpd3*Δ/Δ *rco1*Δ/Δ strain also displayed increased switching frequencies, suggesting that initial induction of switching by Rpd3 is no longer required in the absence of repression by Rco1 and Rpd31 (Fig. 3B).

**FIG 4:**
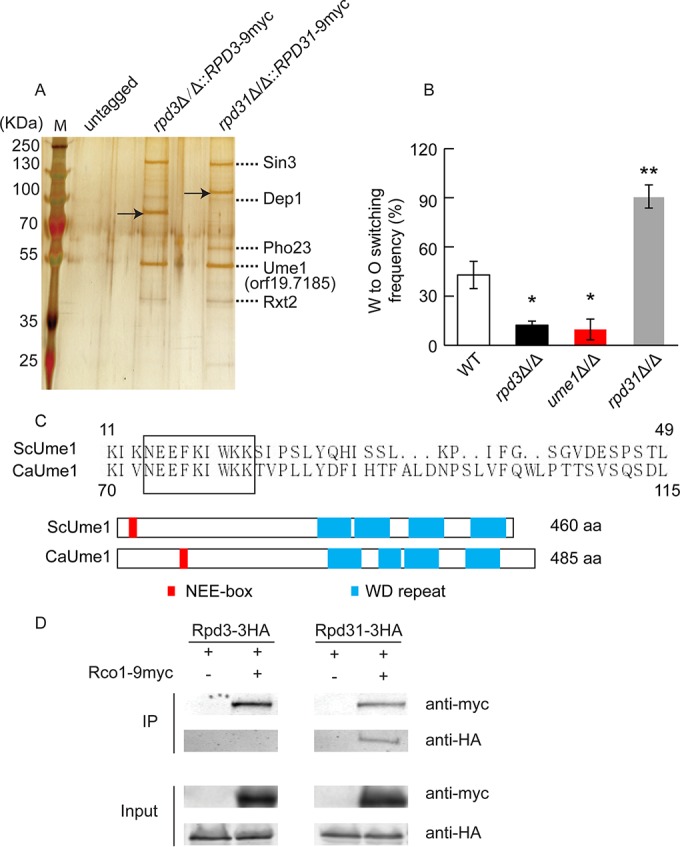
Rpd31 interacts with components of the CaRpd3L and CaRpd31S complexes, whereas Rpd3 is only present in the CaRpd3L complex. (A) Both Rpd3 and Rpd31 interact with components of the putative CaRpd3L complex. Whole-cell extracts were immunoprecipitated with an anti-myc antibody; precipitated proteins were resolved on a 10% SDS-PAGE gel and stained with silver nitrate. Arrows indicate the myc-tagged Rpd3 and Rpd31, respectively. Dotted lines indicate proteins identified by mass spectrometry. (B) W/O switching assays of the WT (J4-2.1) and *rpd3*Δ/Δ, *rpd31*Δ/Δ, and *ume1*Δ/Δ mutant strains on Lee’s GlcNAc medium plus 5% CO_2_. (C) Partial sequence alignment of ScUme1 and orf19.7185 using the ClustalX software (upper part) and their domain organization (lower part). The *C. albicans* orf19.7185 shares both a high homology and conserved domain architecture with ScUme1. We therefore designate the orf19.7185 gene product as Ume1. The rectangular box represents the NEE-box motif. Structural motifs of ScUme1 were retrieved from http://www.uniprot.org/, and orf19.7185 motifs were predicted using the analysis tool Motif Scan (http://myhits.isb-sib.ch/cgi-bin/motif_scan). WD, tryptophan-aspartic acid. (D) Coimmunoprecipitation and mass spectrometry demonstrate physical interactions of Rco1 and Rpd31 but not of Rco1 and Rpd3. Rpd3 and Rpd31 were epitope tagged with 3HA, while 9myc was fused to Rco1 to yield *RCO1*-9myc in heterozygous strain backgrounds *RPD3*/*rpd3*Δ *RCO1*/*rco1*Δ and *RPD31*/*rpd31*Δ *RCO1*/*rco1*Δ, respectively. The same amount of whole-cell extracts from each strain was subjected to immunoprecipitations with an anti-myc antibody and then immunoblotted with anti-HA and c-myc antibodies, respectively. *, *P* < 0.05, and **, *P* < 0.01, compared to the WT strain.

Since Rpd3 and Rpd31 might operate in different complexes, we wanted to test whether Rpd3 is part of a CaRpd3L complex, whereas Rpd31 forms a CaRpd31S complex in *C. albicans*. Therefore, we constructed strains expressing fully functional epitope-tagged Rpd3-9myc and Rpd31-9myc proteins (see Table S5 in the supplemental material) in corresponding heterozygous knockout strains. We then used immunoprecipitation coupled with mass spectrometry to identify possible interaction partners (Fig. 4A). The silver-staining profiles of immunoprecipitable proteins from the *RPD3*-9myc- and *RPD31*-9myc-tagged strains were very similar (Fig. 4A). To identify coprecipitating proteins, the gel bands were cut out and subjected to mass spectrometry, along with proper control samples from the same position in the untagged sample. Indeed, four orthologues of the ScRpd3L complex, Sin3, Dep1, Pho23, and Rxt2, were identified as interaction partners of Rpd3-9myc and Rpd31-9myc. Thus, both Rpd3 and Rpd31 may be part of a CaRpd3L complex.

Interestingly, a hitherto uncharacterized *Candida* protein encoded by orf19.7185 was also identified (Fig. 4A). To test whether orf19.7185 functions in W/O switching, we subjected an *MTL***a**/α homozygous deletion mutant to W/O switching assays. Surprisingly, deletion of orf19.7185 strongly impaired W/O switching compared to the WT control and mimicked the *rpd3*Δ/Δ switching phenotype (Fig. 4B). These data indicated that this protein might act in concert with Rpd3 to regulate W/O switching. Although sequence comparisons indicated some similarities of orf19.7185 to the acetyltransferase subunit Hat2 of *S. cerevisiae* (34), the alignment revealed a high conservation with ScUme1 (Fig. 4C). These conserved domains include the NEE-box motif (NEEFKIWKK) required for the association with ScRpd3 (35) and four predicted tryptophan-aspartic acid (WD) repeats. The high structural similarity and domain organization between orf19.7185 and ScUme1 suggest that they might represent functional orthologues, and we therefore designate the orf19.7185 gene product as Ume1.

Of note, we were unable to identify the putative orthologues Eaf3 and Rco1 present in the Rpd3S complex. Thus, to test for possible physical interactions between Rco1 and Rpd3 or Rpd31, we constructed epitope-tagged strains (Table S5), including the *rpd3*Δ/Δ::*RPD3-*3HA *rco1*Δ/Δ::*RCO1-*9myc and *rpd31*Δ/Δ::*RPD31-*3HA *rco1*Δ/Δ::*RCO1-*9myc strains. Epitope tagging preserved the function and did not alter W/O conversion frequencies (Table S5). Coimmunoprecipitation (co-IP) assays demonstrated that Rco1 physically interacted with Rpd31 but not with Rpd3 (Fig. 4D). These data demonstrate that Rpd31 but not Rpd3 represents the HDAC component of the CaRpd31S regulatory complex in *C. albicans*.

### The divergent C-terminal domain of Rpd31 is important for function.

**FIG 5:**
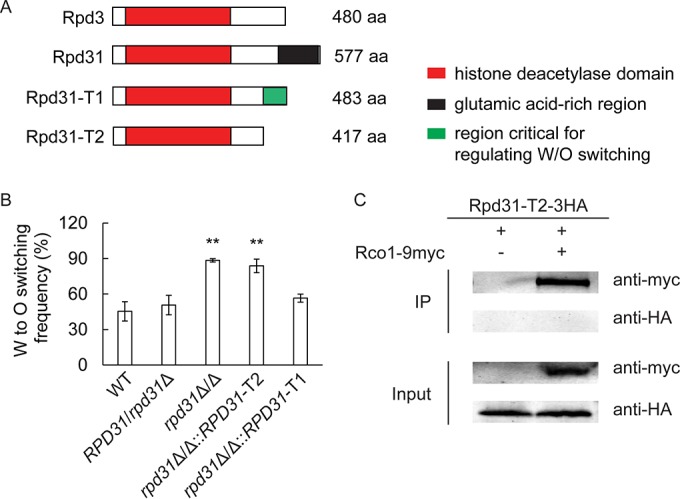
A C-terminal extension domain of Rpd31 is required for W/O switching. (A) Schematic representation of the predicted functional domains of Rpd3, Rpd31, and truncated Rpd31-T1 and Rpd31-T2 variants as predicted by the motif scan tool (http://myhits.isb-sib.ch/cgi-bin/motif_scan). (B) Effects of truncated Rpd31-T1 and Rpd3-T2 variants on the regulation of W/O switching. A long truncated *RPD31* (*RPD31*-T1) and a shorter variant (*RPD31*-T2) were constructed and genomically integrated into the corresponding chromosomal location in *rpd31*Δ/Δ mutants. Correct genomic integration was verified in independent transformants by colony PCR. W/O switching assays were performed on Lee’s GlcNAc medium plus 5% CO_2_. (C) Rpd31-T2 does not interact with Rco1, as shown in a co-IP assay. Rpd31-T2 was epitope tagged with 3HA, and Rco1 was tagged with 9myc in the *RPD31*/*rpd31*Δ heterozygous strain. Co-IP experiments were performed as described in the legend to Fig. 4D. **, *P* < 0.01 compared to the WT strain.

In diploid *C. albicans* cells, *RPD3* and *RPD31* encode 480- and 577-residue proteins, respectively, both of which share highly similar primary sequence with ScRpd3 (433 amino acids [aa]). Rpd3 and Rpd31 share 74% identical residues, but a unique and major difference lies in the C-terminal stretch of about 170 residues, starting from amino acid 407 (Fig. S1). To determine whether the extended C terminus of Rpd31 containing a predicted glutamic acid-rich region (aa 461 to 572 [Fig. 5A]) is required for function in W/O switching, we reintegrated two C-terminally-truncated *RPD31* variants, both still carrying the intact HDAC domain, into the *rpd31*Δ/Δ genome (Fig. 5A). The truncated *RPD31*-T1 encodes a 483-residue protein, which is almost identical in size and domain organization to Rpd3. Furthermore, we constructed a smaller truncated *RPD31*-T2 variant encoding a 417-residue protein lacking all regions differing between Rpd3 and Rpd31. Interestingly, genomic reintegration of Rpd31-T1 fully restored the W/O switching phenotype to wild-type levels (Fig. 5B). Since most of the E-rich region has been deleted in the *RPD31*-T1 variant, this domain might be dispensable for the function of Rpd31 in regulating of W/O switching. In contrast, reintegration of an Rpd31-T2 variant phenocopied *rpd31*Δ/Δ traits in W/O conversion (Fig. 5B). To check for expression levels of Rpd31-T2, we replaced the *RPD31* allele with *RPD31*-T2-3HA in the *RPD31*/*rpd31*Δ strain. Immunoblotting showed that both Rpd31-T2-3HA and Rpd31-3HA were expressed at comparable levels (see Fig. S3 in the supplemental material). This indicates that the region diverging between the two partial Rpd31 variants (residues 418 to 482) is critically required for the function of Rpd31 in modulating W/O switching. Furthermore, to test whether the truncated Rpd31-T2 interacts with Rco1, we constructed a strain coexpressing partial Rpd31-T2-3HA and Rco1-9myc and performed coimmunoprecipitation assays. However, Rpd31-T2 fails to coimmunoprecipitate with Rco1 (Fig. 5C), suggesting that the C terminus may also be required for Rpd31 to associate with Rco1.

### Ectopic *WOR1* bypasses repression of opaque cell formation in *MTL*a/α *rpd3*Δ/Δ cells.

**FIG 6:**
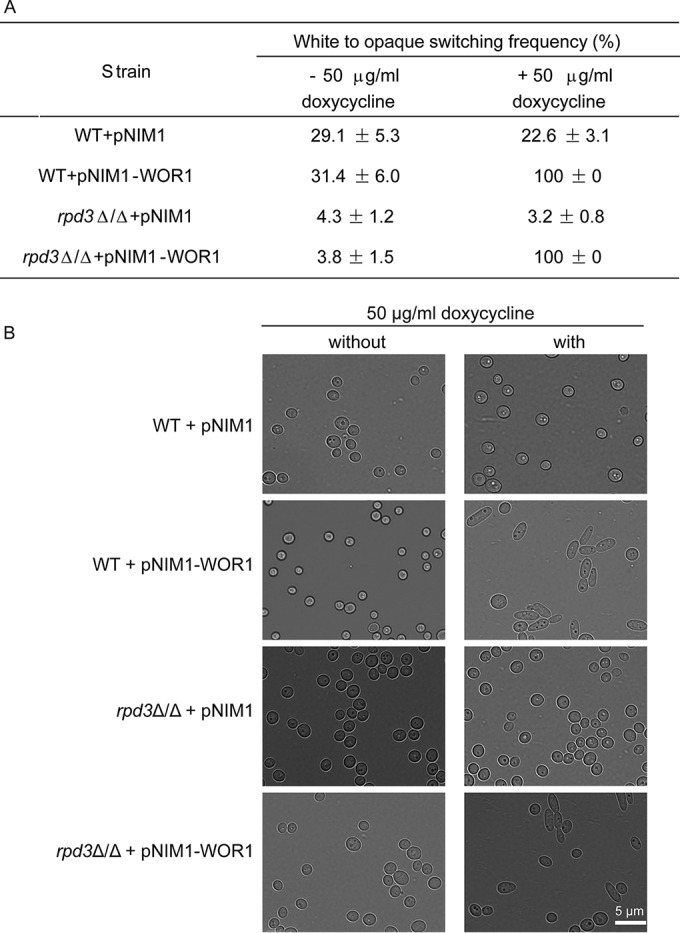
Ectopic *WOR1* expression bypasses repression of the O phase in the absence of Rpd3. All strains were grown on YPD plates at 30°C for 2 days, and then cells were plated on Lee’s GlcNAc medium with or without 50 µg/ml doxycycline, respectively, and incubated at 25°C with 5% CO_2_ for 5 days. W/O switching frequencies were quantified (A) as described and were inspected by light microscopy (B). The scale bar corresponds to 5 µm.

Next, we aimed to unravel how Rpd3 drives W/O switching in *MTL***a**/α strains. In both *MTL* homozygous and heterozygous strains, Wor1 plays a key role in the O cell formation, as well as maintaining O phase stability (12, 25). To study whether ectopic overexpression of Wor1 can bypass the requirement of Rpd3, we expressed *WOR1* under control of a tetracycline-inducible promoter in both WT and *rpd3*Δ/Δ cells (Fig. 6A). Strains containing the empty vector (WT/pNIM1 and *rpd3*Δ/Δ/pNIM1) served as controls. Without induction, 31.4% ± 6.0% of W cells of the WT/pNIM1-WOR1 strain switched to the O phase, whereas the *rpd3*Δ/Δ/pNIN1-WOR1 strain showed 10-fold-lower switching frequencies of about 3.8% ± 1.5%. However, upon induction with 50 µg/ml doxycycline, both the WT/pNIM1-WOR1 and *rpd3*Δ/Δ/pNIM1-WOR1 strains regained 100% W/O conversion. There were no differences in the switching of control strains between inducible and noninducible conditions. Furthermore, cell morphologies upon ectopic *WOR1* expression were verified by light microscopy (Fig. 6B), confirming both W and O phenotypes. In summary, our data indicate that ectopic expression of *WOR1* derepresses O cell conversion in the *rpd3*Δ/Δ strain, strongly suggesting that Rpd3 functions upstream or at least at the level of Wor1.

### Loss of *RPD3* increases acetylation of the *WOR1* promoter and a1/α2 recruitment.

**FIG 7:**
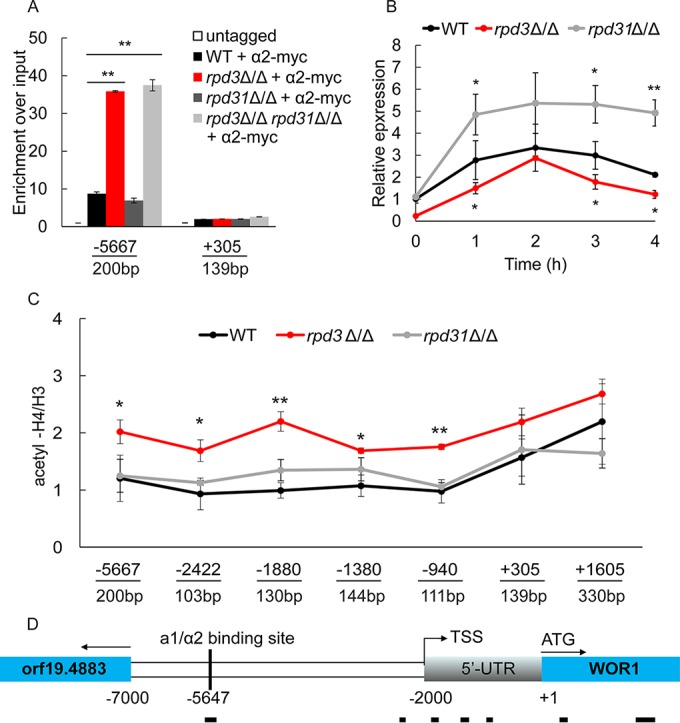
a1**/**α2 binding and H4 acetylation in the *WOR1* promoter in *rpd3*Δ/Δ cells. (A) Deletion of *RPD3* increases binding of the **a**1/α2 repressor to the *cis*-acting site present in the *WOR1* promoter. Binding of myc-tagged α2-myc at the putative **a**1/α2 binding site (bp −5667 to approximately −5467) and in the *WOR1* coding region (bp +304 to approximately +443) was determined in WT and *rpd3*Δ/Δ, *rpd31*Δ/Δ, and *rpd3*Δ/Δ *rpd31*Δ/Δ mutant cells. Whole-cell extracts of α2-myc-tagged strains and the untagged control strain were used for ChIP-qPCR analysis with an anti-myc antibody. The qPCR signals were normalized to an intergenic region on chromosome R. (B) Wor1 expression varies in W cells of the WT, *rpd3*Δ/Δ, and *rpd31*Δ/Δ backgrounds. Overnight cell cultures were diluted to an OD_600_ of 0.5 in fresh Lee’s GlcNAc medium and cultured at 25°C. RNA was isolated at time zero and at 1, 2, 3, and 4 h, and qPCR was performed. Data were normalized to the expression level of the *PAT1* gene. (C) Rpd3 deacetylates the *WOR1* promoter region. Whole-cell extracts from the WT, *rpd3*Δ/Δ, and *rpd31*Δ/Δ strains were subjected to chromatin immunoprecipitation with an antibody against the C terminus of histone H3 and acetylated histone H4. Acetylation levels at the indicated sites were determined by calculating the ratios of the value of acetylated H4 and histone H3 density. (D) Schematic representation of the probes used for the ChIP-qPCR assays. TSS, transcriptional start site; 5′-UTR, 5′-untranslated region. *, *P* < 0.05, and **, *P* < 0.01, compared to the WT strain.

Our data have shown that deletion of *RPD3* in *MTL* homozygous and heterozygous **a**/α strains exerted opposite effects on W/O switching. One possible explanation could be that Rpd3 interacts with different proteins in **a**/α and **a**/**a** cell types. Therefore, we performed immunoprecipitation analysis of *RPD3*-9myc tagged in an **a**/α and **a**/**a** strain background. Silver-staining profiles of immunoprecipitable proteins from both strains were identical (see Fig. S4 in the supplemental material), indicating that Rpd3 interacts and acts with the same protein complex in both **a**/α and **a**/**a** cells. In *MTL***a**/α strains, expression of *WOR1* is efficiently prevented by binding of the heterodimeric **a**1/α2 repressor to a single *cis*-acting motif located at around bp −5647 upstream of the translational start site (14). This repression is fully relieved in *MTL* homozygous strains. Since loss of *RPD3* decreases switching of *MTL***a**/α W to O cells, we assumed that *WOR1* expression is repressed to an even greater extent in *rpd3*Δ/Δ cells compared to WT cells requiring **a**1/α2 complex recruitment. To test our hypothesis, we constructed strains expressing the functional *MTL*α2-myc variant in the WT and *rpd3*Δ/Δ, *rpd31*Δ/Δ, and *rpd3*Δ/Δ *rpd31*Δ/Δ mutant backgrounds (Table S5). We then performed chromatin immunoprecipitation analysis coupled to quantitative PCR (ChIP-qPCR) to verify a possible recruitment of the **a**1/α2 complex to the *WOR1* gene in the W phase. As expected, **a**1/α2 binding to the upstream region of the *WOR1* coding sequence was increased by more than 3-fold in *rpd3*Δ/Δ and *rpd3*Δ/Δ *rpd31*Δ/Δ cells compared to the WT control, while *rpd31*Δ/Δ cells and the WT control showed comparable levels of **a**1/α2 decoration (Fig. 7A). Importantly, **a**1/α2 binding was similar in the coding region of *WOR1*, which served as a negative control. These data strongly indicate that loss of *RPD3* rather than *RPD31* promotes binding of **a**1/α2 to the promoter region of *WOR1*, explaining the enhanced repression of *WOR1* in *rpd3*Δ/Δ cells.

Notably, Wor1 is stochastically expressed at very low levels, with levels varying even among individual cells in the W phase. Wor1 decorates its own DNA regulatory region, forming a positive feedback loop to activate its transcription (36). Therefore, we compared the average steady-state expression of *WOR1* in W phase cells of the WT and *rpd3*Δ/Δ and *rpd31*Δ/Δ mutant backgrounds within one generation to ensure that all the cells remain in the W state. We used a permanent GlcNAc stimulus to increase chances of *WOR1* transcription in individual cells. Preliminary experiments suggested a generation time of about 4 h for cells cultured in Lee’s GlcNAc medium at 25°C (data not shown). Figure 7B shows that expression of *WOR1* in WT within one doubling time first increases and then decreases again. However, while showing expression patterns similar to those in the WT, average *WOR1* levels were significantly reduced in *rpd3*Δ/Δ cells at all time points compared to the WT control. Of note, *rpd31*Δ/Δ cells displayed a distinct *WOR1* expression pattern, showing sustained elevated expression up until 4 h. These data provide strong evidence that the loss of Rpd31 increases the probability of Wor1 to exceed the threshold levels required to activate its positive feedback loop, whereas the converse is true for the *rpd3*Δ/Δ cells. These data are entirely consistent with the observed altered W/O switching frequencies of *rpd3*Δ/Δ and *rpd31*Δ/Δ strains and present a mechanistic explanation for the opposing regulatory phenotypes.

Rpd3 is a putative HDAC deacetylating lysine residues on histones H2A, H2B, H3, and H4. Hence, the histone acetylation status of promoter regions may control the access and affinity of transcription factors to their cognate *cis*-acting DNA sites (37). Therefore, we tested whether the increased binding of **a**1/α2 in *rpd3*Δ/Δ cells can be attributed to higher acetylation levels around the binding site. We used ChIP-qPCR analysis to assess histone acetylation levels at upstream and coding regions of the *WOR1* gene in the WT and *rpd3*Δ/Δ and *rpd31*Δ/Δ mutant strains. Remarkably, deletion of *RPD3* significantly increased the acetylation of histone H4, precisely at the unique putative **a**1/α2 binding site at positions −5667 to −5467, whereas deletion of *RPD31* had no impact at all (Fig. 7C). Furthermore, acetylation at the transcriptional start site at bp −2000 and across the entire 5′-untranslated region (5′-UTR) was also increased in *rpd3*Δ/Δ cells. Interestingly, no significant changes in the acetylation status were detectable in the *WOR1* coding region compared to WT and *rpd31*Δ/Δ cells. Taken together, these data indicate that Rpd3 might be recruited to the promoter region of *WOR1* through a specific CaRpd3L complex, thereby deacetylating the *WOR1* control region and modulating decoration by the **a**1/α2 repressor and possibly other transcription factors.

## DISCUSSION

Eukaryotes possess numerous histone-modifying enzymes to modify nucleosomes and chromatin states to regulate various biological processes in response to environmental stimuli. Here, we show how histone modifications can aid the integration of environmental signals to control W/O morphogenesis in the most prevalent human fungal pathogen, *C. albicans*. We use a systematic reverse-genetics approach by analyzing the phenotypes of 20 homozygous deletion mutants. We identify 6 histone-modifying genes (*DOT1*, *SAS2*, *SET2*, *ELP3*, *HOS1*, and *SPT10*) with specific and hitherto unrecognized roles in W/O switching in *MTL* heterozygous strains. These data demonstrate a novel role for these epigenetic modifiers in the integration of environmental stimuli (GlcNAc and CO_2_) into the cell fate decisions. Notably, we also provide the first evidence for a novel mechanism governing the interplay of epigenetic control and transcriptional repression involving orthologous but functionally distinct HDAC proteins. Specifically, we demonstrate that W/O morphogenesis in *MTL* heterozygous strains of *C. albicans* is critically and inversely controlled by the paralogous HDACs Rpd3 and Rpd31. Remarkably, our data are the first report of two HDACs with the same enzymatic function showing opposing regulatory functions in a single biological process such as W/O morphogenesis.

*C. albicans* harbors two paralogous genes, orf19.2834 (*RPD3*) located on chromosome R and orf19.6801 (*RPD31*) on chromosome III. The genes encode two *RPD3*-like class I HDACs, which are conserved from unicellular yeast cells to humans (37, 38). We show that Rpd3 and Rpd31 of *C. albicans* exert divergent functions in cell fate decisions by virtue of their association with distinct regulatory multiprotein complexes. We identify two Rpd3-related HDAC complexes in *C. albicans*: CaRpd3L and CaRpd31S. While CaRpd3L comprises either Rpd3 or Rpd31 and at least five other subunits (Sin3, Dep1, Pho23, Rxt2, and Ume1), as identified in this study, CaRpd31S includes exclusively Rpd31 and at least one other subunit, Rco1. Remarkably, the CaRpd3L complex drives W/O switching, whereas the CaRpd31S complex acts as a negative regulator of W/O conversion in *C. albicans*.

Gene duplication has long been thought to constitute an evolutionary driving force promoting biodiversity (39), as it leads to either pseudogenes or paralogous genes displaying divergent expression patterns or functions (40). Previous studies mainly addressed the functional diversification of transcription factors, as well as their cognate *cis*-acting motifs (41). Here, we show that the opposing functions of paralogous HDAC genes like *RPD3* and *RPD31* arise from domain-restricted alterations in their coding sequences. *RPD31* inherited all original functions from the ancestral *RPD3*-like mother gene, thus enabling its association with both CaRpd3L and CaRpd31S complexes. *RPD3* may then have undergone a mutational truncation in the C-terminal coding with a selective impact on W/O switching.

Interestingly enough, like *C. abicans*, other diploid *Candida* spp., including *C. dubliniensis*, *C. tropicalis*, and *C. paraposilosis* in the so-called *Candida* clade (42), also possess two yeast-like Rpd3 HDACs. In contrast, all three haploid species (*Candida guilliermondii*, *Candida lusitaniae*, and *Debaryomyces hansenii*) in this clade harbor only one orthologue in their genome, sharing a higher sequence conservation with CaRpd31 rather than CaRpd3. Of note, diploid *Candida* species are more pathogenic than haploid species, and several gene families implicated in pathogenesis are highly enriched for gene duplications (42). Hence, it is tempting to speculate that coevolution with the human host may have triggered gene duplication events, affecting *RPD31* in the diploid *Candida* spp., perhaps to facilitate escape from the immune surveillance or to promote niche colonization. Indeed, W and O cells show restricted niche occupancy, as well as different antifungal drug susceptibilities (10, 12). Hence, host immune surveillance or commensal colonization may have constituted a selective pressure driving the functional diversification of *RPD31*-derived genes in pathogenic *Candida* spp. At this point, it remains unclear how many other biological processes are affected by the *RPD3-*like twins, but host-induced hypha formation and drug resistance may be worthwhile candidates for in-depth investigation in future studies.

We also show that the modulation of W/O switching by Rpd3 requires regulatory genes carried by the *MTL* loci and Wor1, the master regulator of W/O switching. Based on the charge neutralization model (37), we propose that loss of Rpd3 results in hyperacetylated histones in the upstream *WOR1* regulatory region. This increases or facilitates access of the heterodimeric **a**1/α2 repressor and perhaps other as yet unknown regulators to the *WOR1* promoter region. Several transcription factors bind directly to the region upstream of *WOR1* and trigger transcriptional regulation, including the Efg1 repressor binding at around bp −2400 (16) or the Wor4 transcription factor decorating several sites in the *WOR1* upstream region (18). Our data show that the histone acetylation in this region is also elevated in the *rpd3*Δ/Δ strain, implying a possible increase in the binding of Efg1 directly or indirectly. Therefore, removal of *RPD3* in *MTL***a**/α cells may trigger increased recruitment of **a**1/α2 and Efg1 or other regulators such as Wor4, thereby enhancing *WOR1* repression and efficient impairment of W/O switching.

**FIG 8:**
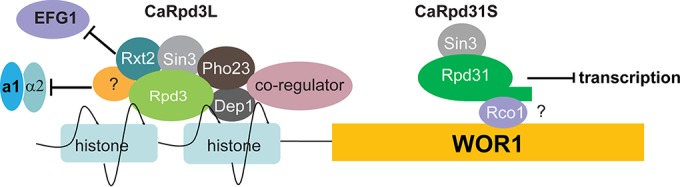
A hypothetical model for the regulation of W/O conversion by Rpd3 and Rpd31. The Rpd3-containing CaRpd3L complex decorates only the upstream region of the *WOR1*, perhaps in cooperation with as yet unknown additional or other coregulators to deacetylate histones. The altered acetylation status regulates affinity or binding of the **a**1/α2 repressor and possibly Efg1, thus promoting *WOR1* transcription. Conversely, the CaRpd31S complex inhibits *WOR1* expression, most likely by decorating the coding region of *WOR1* to inhibit intragenic transcription. The oval with symbol “?” indicates unidentified complex components.

Taken together, our data unequivocally demonstrate distinct and opposing regulatory functions of Rpd3 and Rp31 in W/O switching in *MTL***a**/α heterozygous *C. albicans*. We would like to propose a hypothetical model for the modulation of W/O switching in *MTL***a**/α cells (Fig. 8). Rpd3 binding is significantly associated with increased acetylation of histone H4 in *rpd3*Δ/Δ yeast cells (43). Therefore, based on our data (Fig. 7), we propose that the Rpd3-containing CaRpd3L complex is mainly recruited to the upstream region of *WOR1*, possibly along with as yet unknown coregulators. The increased deacetylation of histones may inhibit binding of negative regulators, eventually driving the stochastic switch from W to O morphostates. Our data are consistent with the fact that yeast ScRpd3L, in association with other coregulators such as Ume6 and Hog1, is exclusively recruited to the promoter regions of target genes to specifically regulate transcription (44, 45). In contrast, ScRpd3S only decorates coding regions and employs Ser5-phosphorylated polymerase II and Set2-methylated histone H3 to suppress intragenic transcription (46). Interestingly, a recent report shows that the dual PHD fingers of ScRco1 recognize the unmodified N terminus of H3, which restricts the recruitment of ScRpd3S to promoter histones normally carrying H3K4me3 marks (47). Of note, CaRco1 also contains two PHD fingers, and all residues critical for ScRco1 function are conserved in CaRco1 (see Fig. S5 in the supplemental material), suggesting that the orthologous CaRco1 and ScRco1 may share similar functions. Together with the results showing that expression of *WOR1* in *rpd31*Δ/Δ W cells is strongly increased compared to that in WT control cells (Fig. 7B), we speculate by analogy that CaRpd31S decoration is restricted to the coding region of *WOR1* to inhibit its intragenic transcription. Unfortunately, and despite intense efforts, we were unable to prove a direct interaction of either Rpd3 or Rpd31 with DNA (data not shown), making chromatin immunoprecipitation sequencing (ChIP-seq) efforts futile and obsolete. This may be caused by high off rates and/or the presence of low-affinity transient complexes or may simply be due to indirect interactions of Rpd31 or Rpd3 with DNA mediated by other complex components. Interestingly enough, we also identify the putative histone acetyltransferase Spt10 as another key regulator of W/O switching, implying that the dynamic interplay of acetylation and deacetylation in the *WOR1* gene controls the stochastic output of Wor1 expression in individual cells. Of note, our data do not indicate a significant increase in the acetylation in the *WOR1* coding region in *rpd31*Δ/Δ cells compared to the WT. However, this may be due to the fact that *WOR1* expression is extremely low in W cells and may in fact not reach detectable levels under normal culture conditions in mixed cell populations. This could also explain why the W morphostate is the default growth phase in *MTL* heterozygous cells. In any case, our date uncover for the first time opposing functions of two HDACs acting in the same biological process. These data exemplify the tight communication and dynamic interplay of epigenetic and transcriptional control in the regulation of developmental cell fate decisions in eukaryotic cells.

## MATERIALS AND METHODS

### Culture conditions.

Strains were routinely grown on YPD (2% Bacto Peptone, 1% yeast extract, 2% dextrose, and 2% Bacto agar) medium. Lee’s glucose medium, Lee’s GlcNAc medium, and synthetic complete (SC) medium used for W/O switching assays were prepared exactly as previously described (48, 49).

### Plasmids and strain constructions.

All strains, plasmids, and primers used in this study are listed in Tables S1, S2, and S3, respectively. A leucine and histidine auxotrophic strain, J4-2.1, was derived from the clinical isolate SZ306 (12) by using plasmids pSFS2A-LEU2 and pSFS2A-HIS1 with the *SAT1*-flipper cassette (50). Single genes of histone modifiers (*DOT1*, *ELP3*, *HDA1*, *HOS1*, *HOS2*, *HOS3*, *HPA2*, *HST1*, *HST2*, *NAT4*, *PHO8*, *PHO13*, *RPD3*, *RPD31*, *SAS2*, *SET1*, *SET2*, *SET3*, *SIR2*, *SPT10*) and *PHO23* and *ROC1* were deleted in strain J4-2.1 (WT) by using the *Candida maltosa* LEU2 (*CmLEU2*) and *Candida dubliniensis* HIS1 (*CdHIS1*) markers as described by Noble and Johnson (51). The same strategy was used to delete *RPD3* and *RPD31* in the spontaneously converted *MTL***a**/**a** strain J130, which is derived from strain J4-2.1. In addition, *RPD3* and *RPD31* genes were also deleted in another clinical *MTL***a**/α strain, JX1250, using the *SAT1* flipper cassette. *RCO1* was deleted in the *rpd3*Δ/Δ and *rpd31*Δ/Δ strains to construct double mutants using the *SAT1* flipper cassette. Likewise, *PHO23* and *RPD31* were also deleted in the *rpd3*Δ/Δ strain to obtain double deletions. All deletion cassettes were constructed by fusion PCR (51) with a fragment containing the selection marker fused to the corresponding upstream and downstream region. Deletion of orf19.7185 in strain J4-2.1 was performed using the linearized plasmid pSFS2A-Ca7185 after digestion with PvuII (34).

For construction of the C-terminal 9myc and 3-hemagglutinin (3HA) tagging cassettes, the 3′ part of the coding sequence and the terminator region of the corresponding gene were fused with the 9myc and the *NAT1* marker amplified from plasmid pFA6a-9myc-NAT1 or 3HA and the *SAT1* flipper cassette from plasmid pFA6a-3HA-SAT1-FLP (34). For the reintegration of *RPD3*, *RPD31*, and truncated *RPD31*-T1as well as partial *RPD31*-T2 variants into the homozygous deletion mutants, the upstream region with the coding sequence and the terminator region were fused with the *SAT1* marker to construct the complementation cassette (15). For the overexpression of *WOR1*, plasmid pNIM1-WOR1 was constructed by subcloning the coding sequence into the BglII-SalI site of plasmid pNIM1 (52). Transformation of *C. albicans* was performed by electroporation as previously described (50). Deletion and integration of genes were confirmed by PCR analysis of genomic DNA.

### W/O switching assays.

The white-opaque (W/O) switching assays were carried out exactly as previously described (12, 15). Briefly, fresh W cells were streaked from 80°C cryo-stocks onto YPD plates and grown at 30°C for 2 days. Cells from single colonies were then plated onto Lee’s glucose medium, Lee’s GlcNAc medium, or SC medium with phloxine B and grown in 5% CO_2_ at 25°C for 5 days. The switching frequency on Lee’s medium was analyzed on day 5. For cells grown on SC medium, pure white cells were replated on fresh SC medium on day 5 and cultured with 5% CO_2_ at 25°C for another 5 days before the switching frequency was analyzed. Pure opaque cells obtained in the W/O switching assays were used to perform the O/W assay using the conditions indicated in the text. At least 350 colonies for each strain were analyzed.

### Immunoprecipitation and mass spectrometry analysis.

Immunoprecipitation experiments were performed as described previously (34), with minor modifications. Cells were grown in 50 ml YPD until they reached an optical density at 600 nm (OD_600_) of 2.0, harvested, washed, and resuspended in 0.5 ml of lysis buffer (10 mM Tris-Cl [pH 8.0], 150 mM NaCl, 0.1% Na-deoxycholate, complete protease inhibitor cocktail [Roche]). Then cells were broken with glass beads (425 to 600 mm [Sigma-Aldrich]) on a FastPrep instrument by shaking 5 times at 6 m/s for 30 s. Whole-cell-free extracts were incubated with protein G-coupled Dynabeads (Invitrogen) containing a covalently attached mouse monoclonal antibody (4A6) recognizing the myc epitope. After washing the beads twice with lysis buffer and once with phosphate-buffered saline (PBS), bound proteins were eluted and resolved on a 10% SDS-PAGE gel followed by silver staining (53) or immunoblotting against myc (4A6) or HA (ab9110 [Abcam]). To identify proteins, bands from different samples were cut at the same height on the silver-stained gel, digested with trypsin, separated on a liquid chromatography (LC) system, and then injected into the mass spectrometer in the mass spectrometry facility at the campus Vienna Biocenter. The raw spectra were matched against the *Candida albicans* database (http://www.candidagenome.org) to identify proteins.

### ChIP-qPCR.

Overnight cultures were diluted to an OD_600_ of 0.15 in fresh YPD medium and then were grown at 30°C to an OD_600_ of 1. Cells were cross-linked with 1% formaldehyde and quenched by 125 mM glycine, and ChIP was carried out essentially as described previously (21). For histone ChIPs, 100 mM sodium butyrate was added to the ChIP lysis buffer. One milligram of whole-cell extract was used for immunoprecipitation. An antibody against the C terminus of histone H3 (ab1791 [Abcam]) was used to detect histone density, and anti-acetyl H4 (06-598 [Millipore]) was used to detect the total acetylated histone H4. *MTL*α*2*-myc ChIP was performed with an antibody against myc (4A6). All ChIP experiments were performed at least with three biological replicates.

For the qPCR quantification of *WOR1* expression, overnight cell cultures were washed and diluted to an OD_600_ of 0.5 in fresh Lee’s GlcNAc medium and cultured at 25°C at 220 rpm in a rotary shaker. Cells were collected at time zero and at 1, 2, 3, and 4 h, respectively. RNA was isolated and qPCR performed as previously described (34). Data were normalized to the expression of *PAT1* using the threshold cycle (ΔΔ*C_T_*) method (34).

## SUPPLEMENTAL MATERIAL

Figure S1  Sequence comparison of *C. albicans* Rpd3 (480 aa) and Rpd31 (577 aa) with *S. cerevisiae* Rpd3 (433 aa). The gene sequences were retrieved from http://www.candidagenome.org and http://www.yeastgenome.org, respectively, and subjected to ClustalX software for alignment. Download Figure S1, TIF file, 10.3 MB

Figure S2  Confirmation of phenotypes of *rpd3*Δ/Δ and *rpd31*Δ/Δ in another clinical strain with a different genetic background (JX1250). Strains were grown on YPD plates at 30°C for 2 days. Then cells were plated on SC-glucose medium at 25°C with 5% CO_2_. After 5 days, cells from white colonies were replated on fresh SC-glucose medium at 25°C with 5% CO_2_ for 5 days. Download Figure S2, TIF file, 3.8 MB

Figure S3  Truncated Rpd31-T2-3HA and Rpd31-3HA are expressed at similar levels. Equivalent amounts of protein extracts prepared from 11 different transformants of the *rpd31*Δ/Δ::*RPD31*-T2-3HA, *rpd31*Δ/Δ:: *RPD31*-3HA, and untagged control cells were fractionated through a 10% SDS-PAGE gel, followed by immunoblotting using an antibody against the HA tag. The solid arrow indicates partial Rpd31-T2-3HA, and the dotted arrow indicates Rpd31-3HA. Download Figure S3, TIF file, 11.6 MB

Figure S4  *RPD3*-9myc-tagged **a**/α and **a**/**a** cells showed the same silver-staining profiles. C-terminally epitope-tagged Rpd3-9myc **a**/α (J126-7) and **a**/**a** (J148-1) strains and the untagged strain J4-2.1 were used for affinity purification of complexes. Immunoprecipitation was performed as described in the legend to Fig. 4A. Download Figure S4, TIF file, 4.4 MB

Figure S5  *C. albicans* Rco1 (688 aa) and *S. cerevisiae* Rco1 (688 aa) share conserved functional domains. (A) Schematic representation of ScRco1 and CaRco1 proteins. The sequences were analyzed with SMART (http://smart.embl-heidelberg.de/smart/set_mode.cgi?NORMAL=1). PHD domains are highlighted in blue, and pink boxes indicate low-complexity regions. (B) Sequence alignment of two PHD fingers. The three conserved residues in PHD1 and PHD2 are highlighted. Download Figure S5, TIF file, 8.6 MB

Table S1  *C. albicans* strains used in this study. Table S1, DOCX file, 0.1 MB

Table S2  Plasmids used in this study. Table S2, DOCX file, 0.1 MB

Table S3  Primers used in this study. Table S3, DOCX file, 0.1 MB

Table S4  Comparison of orthologues involved in Rpd3L and Rpd3S complexes in *S. cerevisiae* and *C. albicans* Table S4, DOCX file, 0.1 MB

Table S5  Epitope tagging does not change the W/O switching of parent strains. Table S5, DOCX file, 0.1 MB
