# Supplementary figures and images for: The Paralogous Histone Deacetylases Rpd3 and Rpd31 Play Opposing Roles in Regulating the White-Opaque Switch in the Fungal Pathogen Candida albicans
Source: mBio. 2016 Nov 15;7(6):e01807-16. doi: 10.1128/mBio.01807-16 (PMC5111407; doi:10.1128/mBio.01807-16)

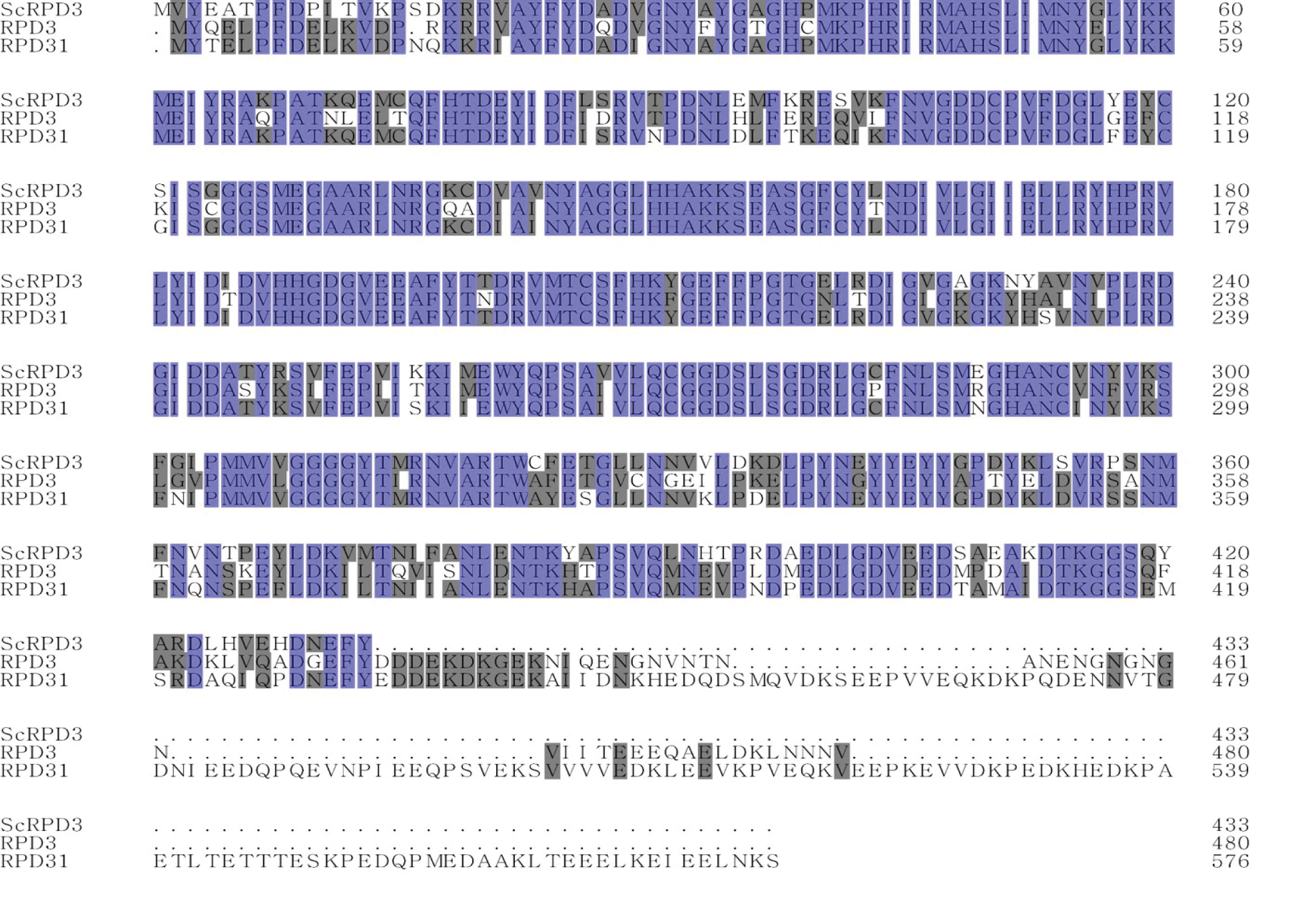

Supplement: Figure S1 — Sequence comparison of C. albicans Rpd3 (480 aa) and Rpd31 (577 aa) with S. cerevisiae Rpd3 (433 aa). The gene sequences were retrieved from http://www.candidagenome.org and http://www.yeastgenome.org, respectively, and subjected to ClustalX software for alignment. Download [file mbo006163061sf1.tif]

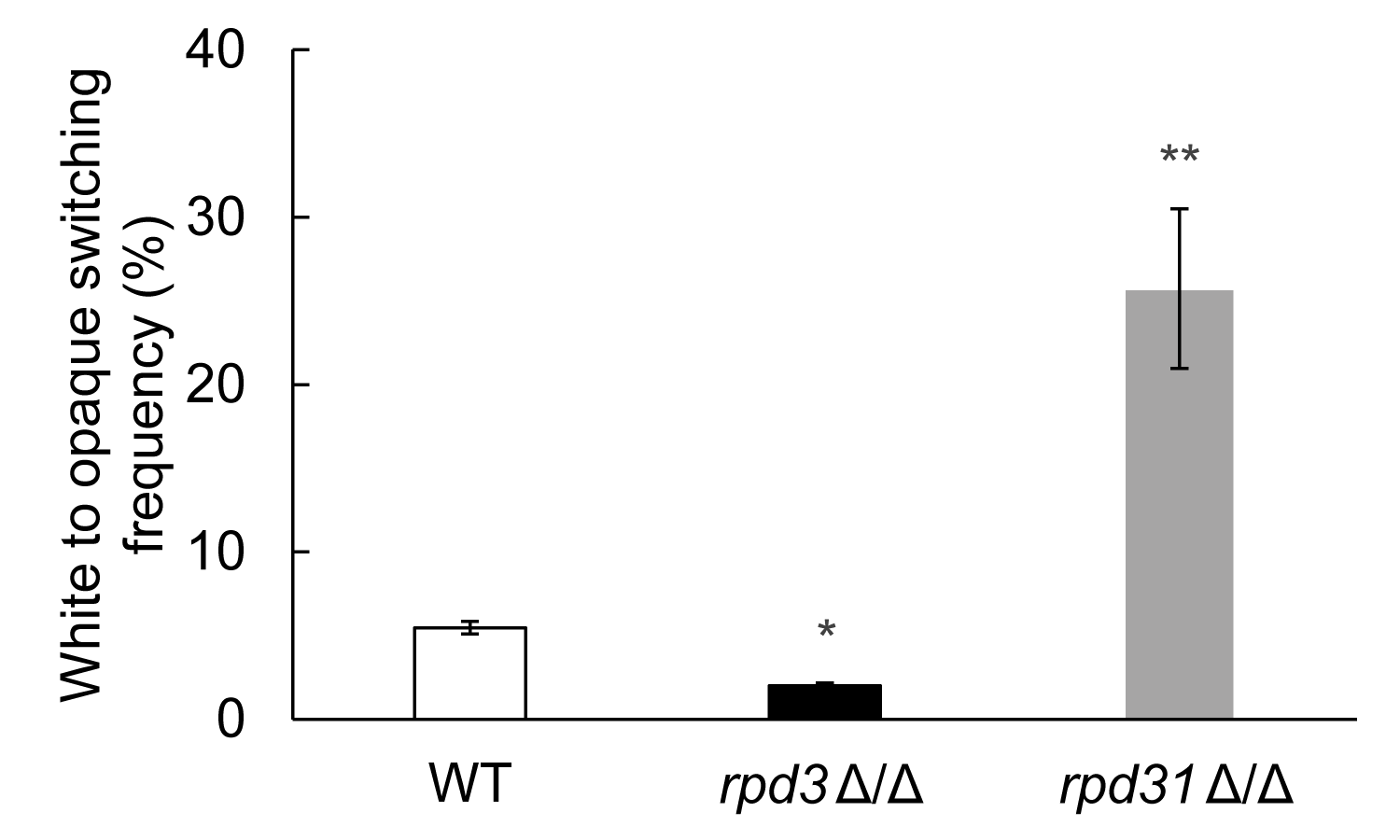

Supplement: Figure S2 — Confirmation of phenotypes of rpd3Δ/Δ and rpd31Δ/Δ in another clinical strain with a different genetic background (JX1250). Strains were grown on YPD plates at 30°C for 2 days. Then cells were plated on SC-glucose medium at 25°C with 5% CO2. After 5 days, cells from white colonies were replated on fresh SC-glucose medium at 25°C with 5% CO2 for 5 days. Download [file mbo006163061sf2.tif]

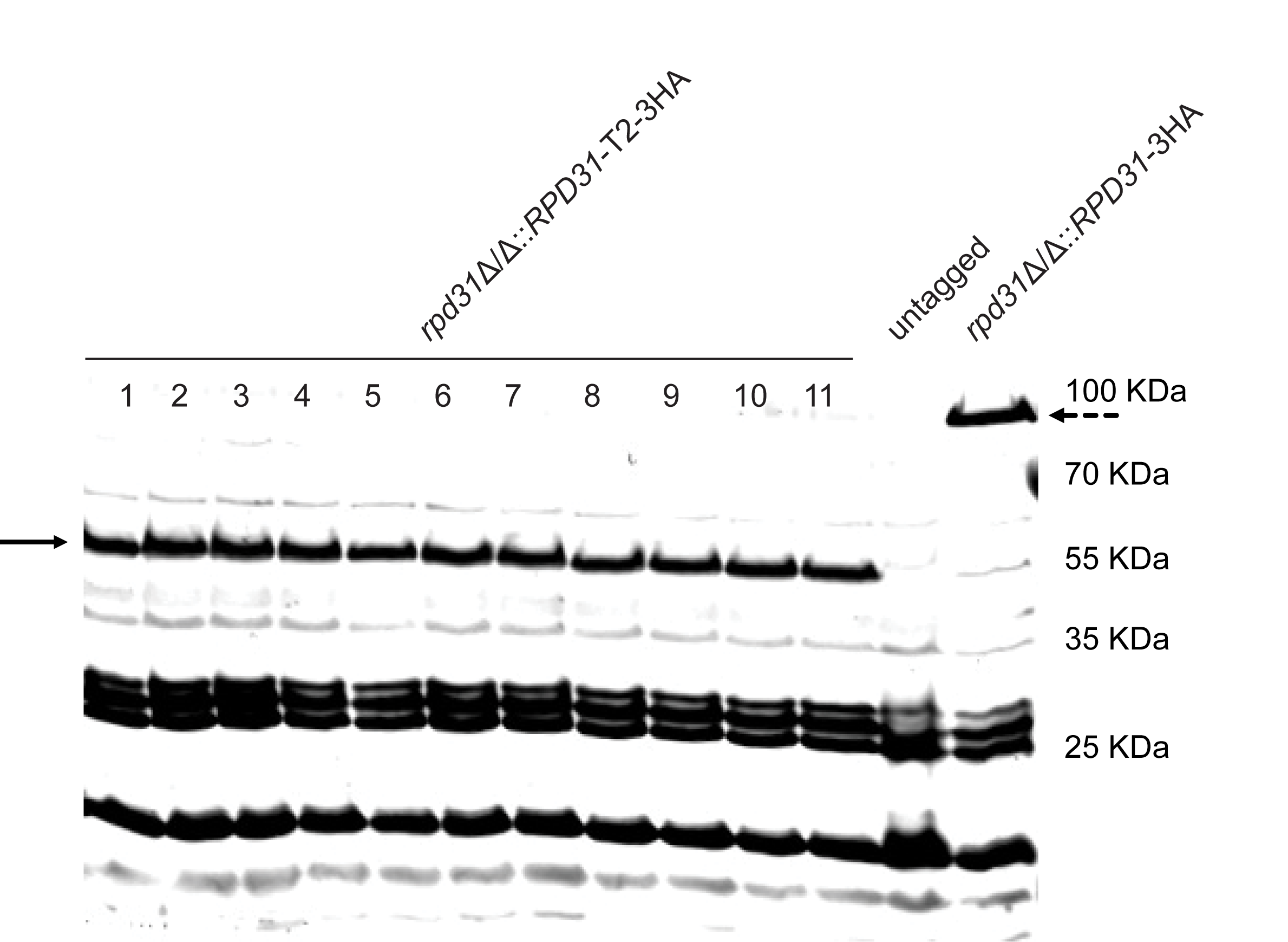

Supplement: Figure S3 — Truncated Rpd31-T2-3HA and Rpd31-3HA are expressed at similar levels. Equivalent amounts of protein extracts prepared from 11 different transformants of the rpd31Δ/Δ::RPD31-T2-3HA, rpd31Δ/Δ:: RPD31-3HA, and untagged control cells were fractionated through a 10% SDS-PAGE gel, followed by immunoblotting using an antibody against the HA tag. The solid arrow indicates partial Rpd31-T2-3HA, and the dotted arrow indicates Rpd31-3HA. Download [file mbo006163061sf3.tif]

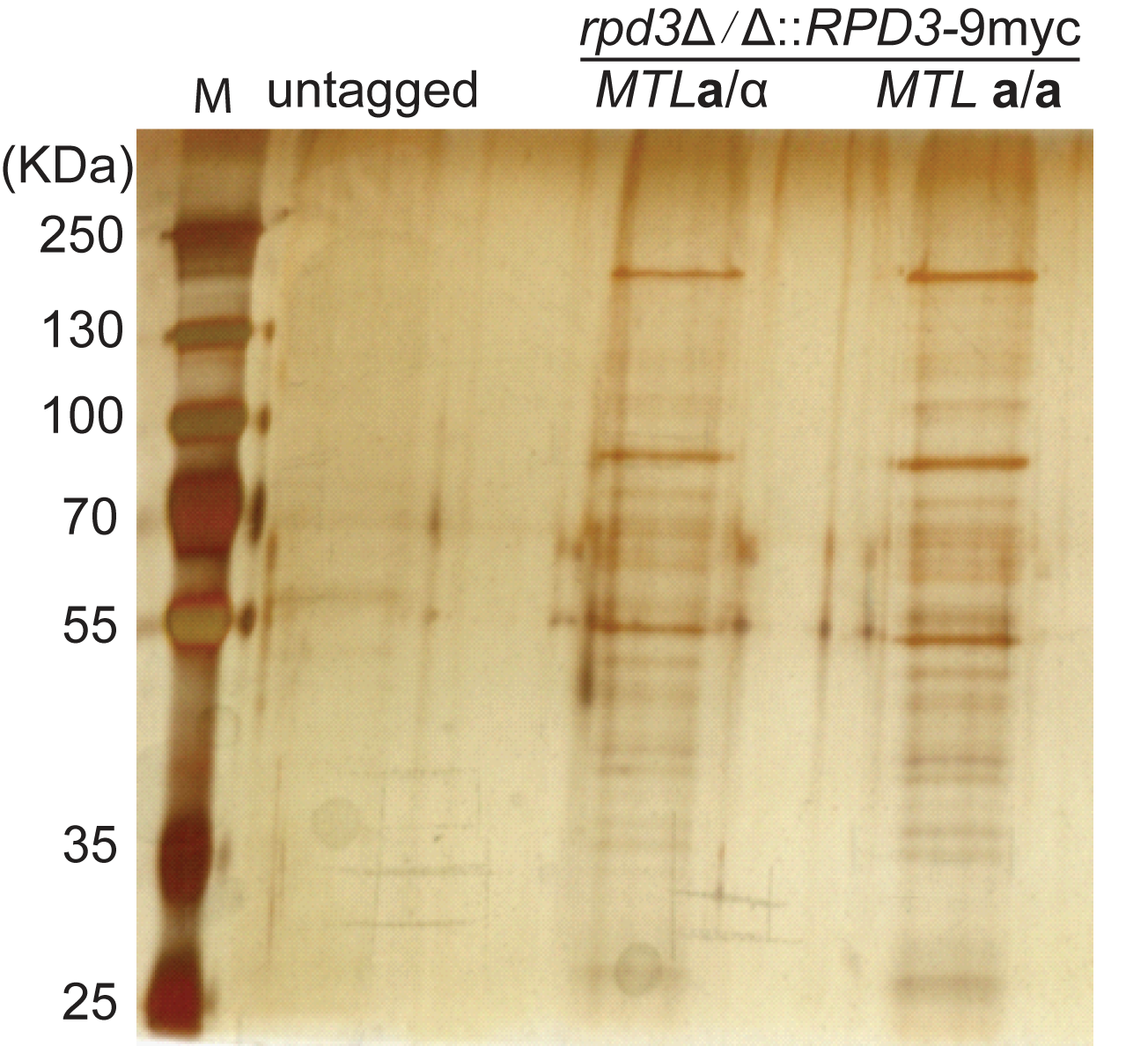

Supplement: Figure S4 — RPD3-9myc-tagged a/α and a/a cells showed the same silver-staining profiles. C-terminally epitope-tagged Rpd3-9myc a/α (J126-7) and a/a (J148-1) strains and the untagged strain J4-2.1 were used for affinity purification of complexes. Immunoprecipitation was performed as described in the legend to Fig. 4A. Download [file mbo006163061sf4.tif]

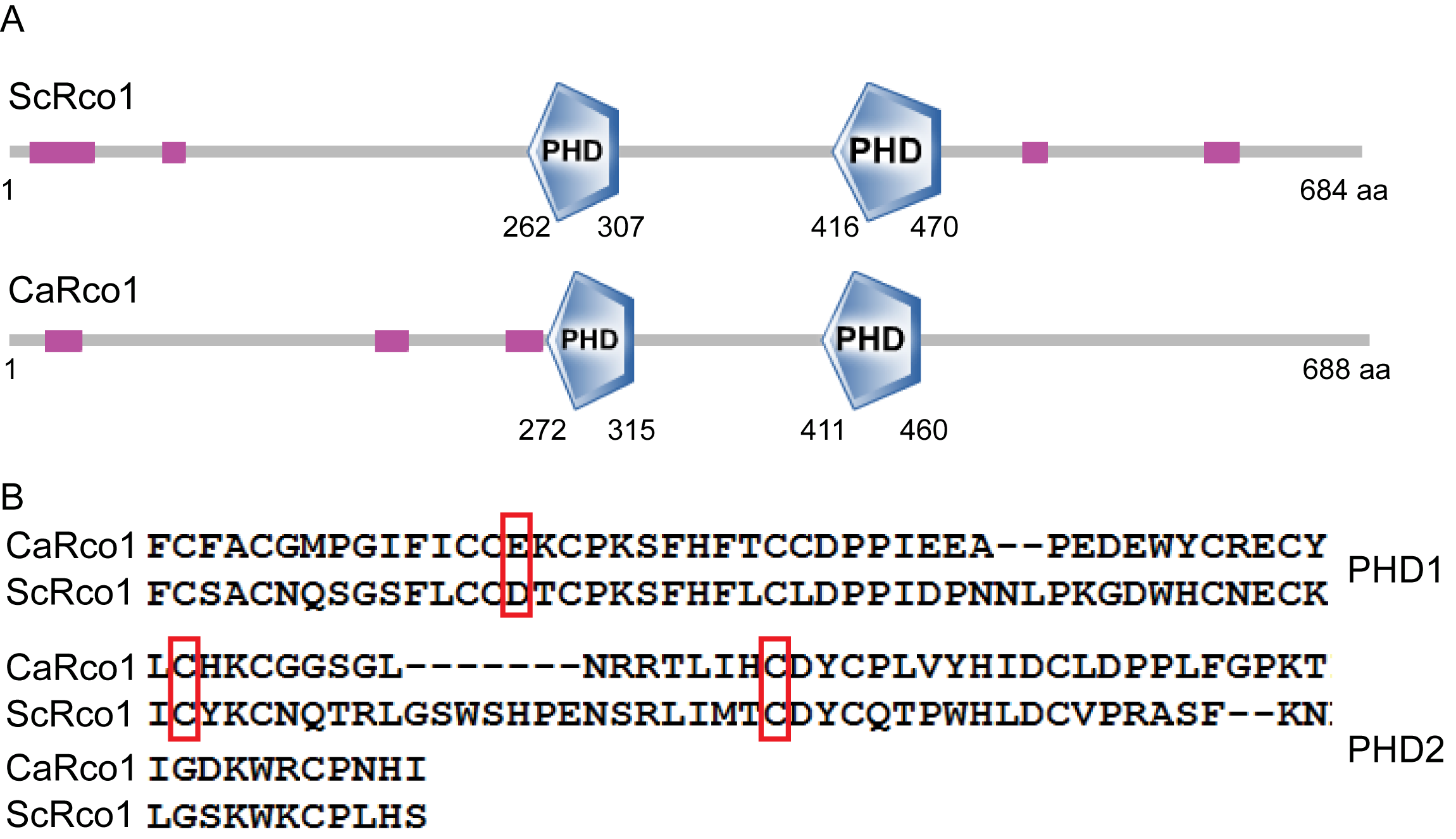

Supplement: Figure S5 — C. albicans Rco1 (688 aa) and S. cerevisiae Rco1 (688 aa) share conserved functional domains. (A) Schematic representation of ScRco1 and CaRco1 proteins. The sequences were analyzed with SMART (http://smart.embl-heidelberg.de/smart/set_mode.cgi?NORMAL=1). PHD domains are highlighted in blue, and pink boxes indicate low-complexity regions. (B) Sequence alignment of two PHD fingers. The three conserved residues in PHD1 and PHD2 are highlighted. Download [file mbo006163061sf5.tif]
