# Supplementary material for: The Paralogous Histone Deacetylases Rpd3 and Rpd31 Play Opposing Roles in Regulating the White-Opaque Switch in the Fungal Pathogen Candida albicans
Source: mBio. 2016 Nov 15;7(6):e01807-16. doi: 10.1128/mBio.01807-16 (PMC5111407; doi:10.1128/mBio.01807-16)
Supplement: Table S1 — C. albicans strains used in this study. [file mbo006163061st1.docx]

Table S1. *C.albicans* strains used in this study.

| Description | Strain | Parent | *MTL* | Genotype | Reference |
| --- | --- | --- | --- | --- | --- |
|  | SZ306 | clinical isolate | **a**/α | wild type | (1) |
| WT (untagged) | J4-2.1 | SZ306 | **a**/α | *leu2*Δ::FRT/l*eu2*Δ::FRT, *his1*Δ::FRT/*his1*Δ::FRT | This study |
| *dot1*Δ/Δ | J39 | J4-2.1 | **a**/α | *dot1*Δ::*C.d.HIS1*/*dot1*Δ::*C.m.LEU2* | This study |
| *elp3*Δ/Δ | J67 | J4-2.1 | **a**/α | *elp3*Δ::*C.d.HIS1*/*elp3*Δ::*C.m.LEU2* | This study |
| *hda1*Δ/Δ | J36 | J4-2.1 | **a**/α | *hda1*Δ::*C.d.HIS1*/*hda1*Δ::*C.m.LEU2* | This study |
| *hos1*Δ/Δ | J42 | J4-2.1 | **a**/α | *hos1*Δ::*C.d.HIS1*/*hos1*Δ::*C.m.LEU2* | This study |
| *hos2*Δ/Δ | J45 | J4-2.1 | **a**/α | *hos2*Δ::*C.d.HIS1*/*hos2*Δ::*C.m.LEU2* | This study |
| *hos3*Δ/Δ | J43 | J4-2.1 | **a**/α | *hos3*Δ::*C.d.HIS1*/*hos3*Δ::*C.m.LEU2* | This study |
| *hpa2*Δ/Δ | J46 | J4-2.1 | **a**/α | *hpa2*Δ::*C.d.HIS1*/*hpa2*Δ::*C.m.LEU2* | This study |
| *hst1*Δ/Δ | J53 | J4-2.1 | **a**/α | *hst1*Δ::*C.d.HIS1*/*hst1*Δ::*C.m.LEU2* | This study |
| *hst2*Δ/Δ | J38 | J4-2.1 | **a**/α | *hst2*Δ::*C.d.HIS1*/*hst2*Δ::*C.m.LEU2* | This study |
| *nat4*Δ/Δ | J52 | J4-2.1 | **a**/α | *nat4*Δ::*C.d.HIS1*/*nat4*Δ::*C.m.LEU2* | This study |
| *pho13*Δ/Δ | J68 | J4-2.1 | **a**/α | *pho13*Δ::*C.d.HIS1*/*pho13*Δ::*C.m.LEU2* | This study |
| *pho8*Δ/Δ | J65 | J4-2.1 | **a**/α | *pho8*Δ::*C.d.HIS1*/*pho8*Δ::*C.m.LEU2* | This study |
| *rpd31*Δ/Δ | J98 | J4-2.1 | **a**/α | *rpd31*Δ::*C.d.HIS1*/*rpd31*Δ::*C.m.LEU2* | This study |
| *rpd3*Δ/Δ | J64 | J4-2.1 | **a**/α | *rpd3*Δ::*C.d.HIS1*/*rpd3*Δ::*C.m.LEU2* | This study |
| *sas2*Δ/Δ | J66 | J4-2.1 | **a**/α | *sas2*Δ::*C.d.HIS1*/*sas2*Δ::*C.m.LEU2* | This study |
| *set1*Δ/Δ | J50 | J4-2.1 | **a**/α | *set1*Δ::*C.d.HIS1*/*set1*Δ::*C.m.LEU2* | This study |
| *set2*Δ/Δ | J48 | J4-2.1 | **a**/α | *set2*Δ::*C.d.HIS1*/*set2*Δ::*C.m.LEU2* | This study |
| *set3*Δ/Δ | J71 | J4-2.1 | **a**/α | *set3*Δ::*C.d.HIS1*/*set3*Δ::*C.m.LEU2* | This study |
| *sir2*Δ/Δ | J69 | J4-2.1 | **a**/α | *sir2*Δ::*C.d.HIS1*/*sir2*Δ::*C.m.LEU2* | This study |
| *spt10*Δ/Δ | J70 | J4-2.1 | **a**/α | *spt10*Δ::*C.d.HIS1*/*spt10*Δ::*C.m.LEU2* | This study |
| *rpd3*Δ/Δ*::RPD3* | J166-2 | J64 | **a**/α | *rpd3*Δ::*C.d.HIS1*/*rpd3*Δ::*RPD3-SAT1* | This study |
| *rpd31*Δ/Δ*::RPD31* | J152-8 | J98 | **a**/α | *rpd31*Δ::*C.m.LEU2*/*rpd31*Δ::*RPD31-SAT1* | This study |
| WT | J130 | J4-2.1 | **a**/**a** | *leu2*Δ::FRT/l*eu2*Δ::FRT, *his1*Δ::FRT/*his1*Δ::FRT | This study |
| *rpd3*Δ/Δ | J132 | J130 | **a**/**a** | *rpd3*Δ::*C.d.HIS1*/*rpd3*Δ::*C.m.LEU2* | This study |
| *rpd31*Δ/Δ | J134 | J130 | **a**/**a** | *rpd31*Δ::*C.d.HIS1*/*rpd31*Δ::*C.m.LEU2* | This study |
| *pho23*Δ/Δ | J109 | J4-2.1 | **a**/α | *pho23*Δ::*C.d.HIS1*/*pho23*Δ::*C.m.LEU2* | This study |
| *rco1*Δ/Δ | J110 | J4-2.1 | **a**/α | *rco1*Δ::*C.d.HIS1*/*rco1*Δ::*C.m.LEU2* | This study |
| *rpd3*Δ/Δ *rco1*Δ/Δ | J120 | J64 | **a**/α | *rpd3*Δ::*C.d.HIS1*/*rpd3*Δ::*C.m.LEU2*, *rco1*Δ::FRT/*rco1*Δ::*SAT1* | This study |
| *rpd31*Δ/Δ *rco1*Δ/Δ | J121-11 | J98 | **a**/α | *rpd31*Δ::*C.d.HIS1*/*rpd31*Δ::*C.m.LEU2*, *rco1Δ:*:FRT/*rco1*Δ::*SAT1* | This study |
| *rpd3*Δ/Δ pho23Δ/Δ | J125 | J64 | **a**/α | *rpd3*Δ::*C.d.HIS1*/*rpd3*Δ::*C.m.LEU2*, *pho23*Δ::FRT/*pho23*Δ::*SAT1* | This study |
| *rpd3*Δ/Δ *rpd31*Δ/Δ | J129 | J64 | **a**/α | *rpd3*Δ::*C.d.HIS1*/*rpd3*Δ::*C.m.LEU2*, *rpd31*Δ::FRT/*rpd31*Δ::*SAT1* | This study |
| *RPD3/rpd3*Δ | J54 | J4-2.1 | **a**/α | *RPD3*/*rpd3*Δ::*C.m. LEU2* | This study |
| *rpd3*Δ/Δ::*RPD3-*9myc | J126-7 | J54 | **a**/α | *rpd3*Δ::*C.m. LEU2*/*rpd3*Δ::*RPD3-*9myc-NAT1 | This study |
| *RPD31/rpd31*Δ | J22 | J4-2.1 | **a**/α | *RPD31*/*rpd31*Δ::*C.m. LEU2* | This study |
| *rpd31*Δ/Δ::*RPD31-*9myc | J127-6 | J22 | **a**/α | *rpd31*Δ::*C.m. LEU2*/*rpd31*Δ::*RPD31-*9myc-NAT1 | This study |
| *ume1*Δ/Δ | J141 | J4-2.1 | **a**/α | *ume1*Δ::FRT/*ume1*Δ::*SAT1* | This study |
| Rpd3-3HA | J178-8 | J54 | **a**/α | *rpd3*Δ::*C.m. LEU2*/*rpd3*Δ::*RPD3*-3HA-FRT, *RCO1*/*rco1*Δ::*C.d.HIS1* | This study |
| Rpd3-3HA Rco1-9myc | J180-6 | J178-8 | **a**/α | *rpd3*Δ::*C.m. LEU2*/*rpd3*Δ::*RPD3*-3HA-FRT, *rco1*Δ::*C.d.HIS1*/*rco1*Δ::*RCO1*-9myc-NAT1 | This study |
| Rpd31-3HA (*rpd31*Δ/Δ:: *RPD31*-3HA) | 179-8.1 | J22 | **a**/α | *rpd31*Δ::*C.m. LEU2*/*rpd31*Δ::*RPD31*-3HA-FRT, *RCO1*/*rco1*Δ::FRT | This study |
| Rpd31-3HA Rco1-9myc | J181-1 | 179-8.1 | **a**/α | *rpd31*Δ::*C.m. LEU2*/*rpd31*Δ::*RPD31*-3HA-FRT, *rco1*Δ::FRT/*rco1*Δ::*RCO1*-9myc-NAT1 | This study |
| *rpd31*Δ/Δ*::RPD31*-T2 | J176-2 | J98 | **a**/α | *rpd31*Δ::*C.d.HIS1*/*rpd31*Δ::*RPD31-*T2*-SAT1* | This study |
| *rpd31*Δ/Δ*::RPD31*-T1 | J177-1 | J98 | **a**/α | *rpd31*Δ::*C.m.LEU2*/*rpd31*Δ::*RPD31-*T1*-SAT1* | This study |
| Rpd31-T2-3HA | J184-2.1 | J22 | **a**/α | *rpd31*Δ::*C.m. LEU2*/*rpd31*Δ:: *RPD31*-T2-3HA-FRT | This study |
| Rpd31-T2-3HA Rco1-9myc | J185-1 | J184-2.1 | **a**/α | *rpd31*Δ::*C.m. LEU2*/*rpd31*Δ::*RPD31*-T2-3HA-FRT, *RCO1*/*rco1*Δ::*RCO1*-9myc-NAT1 | This study |
| WT + pNIM1 | J111 | J4-2.1 | **a**/α | +pNIM1 | This study |
| WT + pNIM1-WOR1 | J112 | J4-2.1 | **a**/α | +PNIM1-WOR1 | This study |
| *rpd3*Δ/Δ + pNIM1 | J113 | J64 | **a**/α | +pNIM1 | This study |
| *rpd3*Δ/Δ + pNIM1-WOR1 | J114-4 | J64 | **a**/α | +PNIM1-WOR1 | This study |
| WT + α2-myc | J161-2 | J4-2.1 | **a**/α | *leu2*Δ::FRT/*leu2*Δ::FRT, *his1*Δ::FRT/*his1*Δ::FRT, *MTL*α2Δ::*MTL*α2*-*9myc-NAT1 | This study |
| *rpd3*Δ/Δ + α2-myc | J157-6 | J64 | **a**/α | *rpd31*Δ::*C.d.HIS1*/*rpd31*Δ::*C.m.LEU2*, *MTL*α2Δ::*MTL*α2-9myc-NAT1 | This study |
| *rpd31*Δ/Δ + α2-myc | J171-1 | J98 | **a**/α | *rpd3*Δ::*C.d.HIS1*/*rpd3*Δ::*C.m.LEU2*, *MTL*α2Δ::*MTL*α2-9myc-NAT1 | This study |
| *rpd3*Δ/Δ *rpd31*Δ/Δ + α2-myc | J172-1 | J129 | **a**/α | *rpd3*Δ::*C.d.HIS1*/*rpd3*Δ::*C.m.LEU2*, *rpd31*Δ::FRT/*rpd31*Δ::FRT, *MTL*α2Δ::*MTL*α2-9myc-NAT1 | This study |
| WT | JX1250 | clinical isolate | **a**/α | wild type | (1) |
| *rpd3*Δ/Δ | J123 | JX1250 | **a**/α | *rpd3*Δ::FRT/*rpd3*Δ::*SAT1* | This study |
| *rpd31*Δ/Δ | J156 | JX1250 | **a**/α | *rpd31*Δ::FRT/*rpd31*Δ::*SAT1* | This study |
| *rpd3*Δ/Δ*::RPD3-*9myc | J148-1 | J130 | **a**/**a** | *rpd3*Δ::*C.m. LEU2*/*rpd3*Δ::*RPD3-*9myc-NAT1 | This study |
| *rpd31*Δ/Δ::*RPD31*-T2-3HA | J183 | J22 | **a**/α | *rpd31*Δ::*C.m. LEU2*/*rpd31*Δ:: *RPD31*-T2-3HA-*SAT1*-FLP | This study |

1. **Xie J, Tao L, Nobile CJ, Tong Y, Guan G, Sun Y, Cao C, Hernday AD, Johnson AD, Zhang L, Bai FY, Huang G.** 2013. White-opaque switching in natural MTLa/alpha isolates of *Candida albicans*: evolutionary implications for roles in host adaptation, pathogenesis, and sex. PLoS Biol **11:**e1001525.
