# Supplementary material for: The Paralogous Histone Deacetylases Rpd3 and Rpd31 Play Opposing Roles in Regulating the White-Opaque Switch in the Fungal Pathogen Candida albicans
Source: mBio. 2016 Nov 15;7(6):e01807-16. doi: 10.1128/mBio.01807-16 (PMC5111407; doi:10.1128/mBio.01807-16)
Supplement: Table S2 — Plasmids used in this study. [file mbo006163061st2.docx]

Table S2. Plasmids used in this study.

| Name | Parent | Enzymes used for linearization | Purpose | Reference |
| --- | --- | --- | --- | --- |
| pSFS2A |  |  | construct the reconsituted strains | (1) |
| pSFS2A-LEU2 | pSFS2A | *Apa*I and *Sac*I | knockout  *LEU2* | This study |
| pSFS2A-HIS1 | pSFS2A | *Apa*I and *Sac*I | knockout the first copy of *HIS1* | This study |
| pSFS2A-HIS1-2 | pSFS2A | *Apa*I and *Sac*I | knockout the sceond copy of *HIS1* | This study |
| pSN40 |  |  | amplification of *C.m.LEU2* marker | (2) |
| pSN52 |  |  | amplification of *C.d.HIS1* marker | (2) |
| pSFS2A-Ca7185 | pSFS2A | *Pvu*II | knockout orf19.7185 | (3) |
| pFA6a-9myc-NAT1 | pFA6a-3HA-NAT1 |  | 9myc tagging | (3) |
| pFA6a-3HA-SAT1-FLP | pFA6a-3HA-NAT1 |  | 3HA tagging | (3) |
| pNIM1 |  | *Apa*I | empty vector as a control | (4) |
| pNIM1+WOR1 | pNIM1 | *Apa*I | ectopic expression of *WOR1* | This study |

1. **Reuss O, Vik A, Kolter R, Morschhauser J.** 2004. The SAT1 flipper, an optimized tool for gene disruption in *Candida albicans*. Gene **341:**119-127.

2. **Noble SM, Johnson AD.** 2005. Strains and Strategies for Large-Scale Gene Deletion Studies of the Diploid Human Fungal Pathogen *Candida albicans*. Eukaryotic Cell **4:**298-309.

3. **Tscherner M, Stappler E, Hnisz D, Kuchler K.** 2012. The histone acetyltransferase Hat1 facilitates DNA damage repair and morphogenesis in *Candida albicans*. Mol Microbiol **86:**1197-1214.

4. **Park YN, Morschhauser J.** 2005. Tetracycline-inducible gene expression and gene deletion in *Candida albicans*. Eukaryot Cell **4:**1328-1342.
