# Supplementary material for: The Paralogous Histone Deacetylases Rpd3 and Rpd31 Play Opposing Roles in Regulating the White-Opaque Switch in the Fungal Pathogen Candida albicans
Source: mBio. 2016 Nov 15;7(6):e01807-16. doi: 10.1128/mBio.01807-16 (PMC5111407; doi:10.1128/mBio.01807-16)
Supplement: Table S3 — Primers used in this study. [file mbo006163061st3.docx]

Table S3 Primers used in this study.

| Plamids construction | | |
| --- | --- | --- |
| Name | Sequence (5' - 3') | Reference |
| LEU2apaF | aatacaGGGCCCTCTCAATACCCTGGTTGCGT | This study |
| LEU2xhoR | aatacaCTCGAGAATCACAACCTCATGCTCGC | This study |
| LEU2sacIIF | aatacaCCGCGGTGCACACAACTGAGTTACCG | This study |
| LEU2sacIR | aatacaGAGCTCCGCTACCCAACAAAGAGACC | This study |
| HIS1apaF | aatacaGGGCCCAACAGTGTCGCCAGAATGTG | This study |
| HIS1xhoR | aatacaCTCGAGTTTCAACGAAATGGCCTCCC | This study |
| HIS1sacIIF | aatacaCCGCGGACCATCTTCGACCGTCATGT | This study |
| HIS1sacIR | aatacaGAGCTCAAGTATCTGACGGCACCCTT | This study |
| HIS1xhoR2 | aatacaCTCGAGTCCATTATCGGTAGTTGGTGGT | This study |
| WOR1SalIF | aatcttGTCGACATGTCTAATTCAAGTATAGTCCC | This study |
| WOR1BglIIR | aatcttAGATCTCTAAGTACCGGTGTAATACG | This study |
| Gene deletion cassettes based on fusion PCR strategy | | |
| Name | Sequence (5' - 3') | Reference |
| M5 | ccgctgctaggcgcgccgtgACCAGTGTGATGGATATCTGC | (1) |
| M3 | gcagggatgcggccgctgacAGCTCGGATCCACTAGTAACG | (1) |
| 5-FRT-FLP-SAT | ccgctgctaggcgcgccgtgTACCGGGCCCCCCCTCGAGGAAGTT | This study |
| 3-FRT-FLP-SAT | gcagggatgcggccgctgacGCCGCTCTAGAACTAGTGGATC | This study |
| 55_CA1759 | ACACAATCCAGCTTACCACAG | This study |
| 53_CA1759 | cacggcgcgcctagcagcggCTGTTGTGTCGGTCGGTTAA | This study |
| 35_CA1759 | gtcagcggccgcatccctgcGCAATCAACACAGCCTTTGG | This study |
| 33_CA1759 | CTGTGTGTGTGTGGCAATGA | This study |
| 55_CA6506 | AGTCCTGAGTTTGATGCCGA | This study |
| 53_CA6506 | cacggcgcgcctagcagcggATGTTCAGCAATGGAAAACCC | This study |
| 35_CA6506 | gtcagcggccgcatccctgcTCCTCAGTCAACTTTCTAGCCT | This study |
| 33_CA6506 | AGTGCCCATCATGCTGACTA | This study |
| 55_CA2834 | TCTTCCAATGAATGGGTAGACC | (2) |
| 53_CA2834 | cacggcgcgcctagcagcggCATTTTTTCTTCGGTTGGTTTG | (2) |
| 35_CA2834 | gtcagcggccgcatccctgcTGAATGGCAAATAATGTAGATAGAAG | (2) |
| 33_CA2834 | TCTCAATATGTCAAACCATGTGG | (2) |
| 55_CA6801 | AATAACAGTTACTGTCACCGCC | (2) |
| 53_CA6801 | cacggcgcgcctagcagcggCATGGTGGACGAGTTTGGTTG | (2) |
| 35_CA6801 | gtcagcggccgcatccctgcTAAAAGTTCATAAATAAAAGGATATTTAGATTG | (2) |
| 33_CA6801 | TCCGAAGGGAATTTAATTGG | (2) |
| 55_CA1755 | GATCTTGAAGAAGTAATGTTTCCTG | (2) |
| 53_CA1755 | cacggcgcgcctagcagcggCATGATTTGTTAAATGAGTGTTTATC | (2) |
| 35_CA1755 | gtcagcggccgcatccctgcTAGATATTTATTTTGTTCATACAAGAAAAA | (2) |
| 33_CA1755 | GTTTCTGCTGAAGAAGCCG | (2) |
| 55_CA1992 | ATCACAACAGTAGGGCATCAAC | (2) |
| 53_CA1992 | cacggcgcgcctagcagcggCATTACTAGAGGATTTCTCTCAAATAAC | (2) |
| 35_CA1992 | gtcagcggccgcatccctgcTGATTAAATTAATATTGGTGTCTTTAATG | (2) |
| 33_CA1992 | TCAAGATCAACAATATGTGGTGG | (2) |
| 55_CA2087 | AAGGCAAGAAGAGAATCTTTGG | (2) |
| 53_CA2087 | cacggcgcgcctagcagcggCATGCATGAACTTTTATGATATTAGATG | (2) |
| 35_CA2087 | gtcagcggccgcatccctgcTGAATTAGTTACATACTTTTTTTTTTTAAA | (2) |
| 33_CA2087 | ACATGGAATACGTTGACTGGG | (2) |
| 55_CA2361 | TCTTCATCGTAATACGACGGTC | (2) |
| 53_CA2361 | cacggcgcgcctagcagcggCATGATCTATTTAAAGATTGTAAGGAAT | (2) |
| 35_CA2361 | gtcagcggccgcatccctgcTAGTTAAAAAGATCACACACTTACTGTTG | (2) |
| 33_CA2361 | TGATTTCGATCAAAGAGCTGC | (2) |
| 55_CA2580 | CATTCGAGCTAAACGAAGCTC | (2) |
| 53_CA2580 | cacggcgcgcctagcagcggCATGGTTTAAAGTTATAGTCTGTTGTAAG | (2) |
| 35_CA2580 | gtcagcggccgcatccctgcTTAAACTGTCTCAACAAAAATATAATATAC | (2) |
| 33_CA2580 | GCCTTCATAAAACGTTCTCTCAC | (2) |
| 55_CA2606 | GTGTGAGTAGAGTTCACAACAAGTG | (2) |
| 53_CA2606 | cacggcgcgcctagcagcggCATTCTTAAAAAGGAAAATGATAATAG | (2) |
| 35_CA2606 | gtcagcggccgcatccctgcTGATTCGAGTAGAAACAACAACAAC | (2) |
| 33_CA2606 | CAACACTTTCCAAACACTCTCAG | (2) |
| 55_CA2772 | CAATGGAAATATTGTTACAGATGG | (2) |
| 53_CA2772 | cacggcgcgcctagcagcggCATTATAAAAAACTTCCCCAAGTAA | (2) |
| 35_CA2772 | gtcagcggccgcatccctgcTAGAGTATATTAGAACATGAAATGTATATATATAAG | (2) |
| 33_CA2772 | AACAACTACAACAACTACTACTACTCCAC | (2) |
| 55_CA4411 | TGCCAGTAAACGAGGTTACG | (2) |
| 53_CA4411 | cacggcgcgcctagcagcggCATTAATCTATTAACAATAATACAAACCAG | (2) |
| 35_CA4411 | gtcagcggccgcatccctgcTAAAATTATATATCTACCACTAAACTGTTTAAG | (2) |
| 33_CA4411 | TTGTTACTGGAAAGGGACCC | (2) |
| 55_CA4444 | AGATAAAGAGTTGTTCTCTCAGTTCC | (2) |
| 53_CA4444 | cacggcgcgcctagcagcggCATTAGAAACGGAATGTATTGGG | (2) |
| 35_CA4444 | gtcagcggccgcatccctgcTAGAAACATATGCCTTGCTATTTTT | (2) |
| 33_CA4444 | TGCAAGCAAAACTAAGAACGTC | (2) |
| 55_CA4664 | TTTATTGGTTCTCAATGGCAG | (2) |
| 53_CA4664 | cacggcgcgcctagcagcggCATTGTTGTTTGTGTTGGCA | (2) |
| 35_CA4664 | gtcagcggccgcatccctgcTAAGTATTTGTGTAGAACATTCTTCCC | (2) |
| 33_CA4664 | CAACAAATGGGTAGCAAGGTC | (2) |
| 55_CA4736 | CCTATCTTTACACTACTGTTCACCG | (2) |
| 53_CA4736 | cacggcgcgcctagcagcggCATGATGGAATATGTAGCAATAAAAAG | (2) |
| 35_CA4736 | gtcagcggccgcatccctgcTAAGTGTGTATGTACATATACCAAGAAC | (2) |
| 33_CA4736 | ACAATGAAGCAAGTGAATAACG | (2) |
| 55_CA4761 | CACCCTTTCCTCTATTCTTTGC | (2) |
| 53_CA4761 | cacggcgcgcctagcagcggCATTATTGATTGTTATTTGTTATTAGTAATAATTG | (2) |
| 35_CA4761 | gtcagcggccgcatccctgcTGAAGAGCCAAAACAAGATAAAG | (2) |
| 33_CA4761 | TATTGCTGCTTATAATGTATAGGGAG | (2) |
| 55_CA5377 | CAGAATCTTGACCTGTGATTCC | (2) |
| 53_CA5377 | cacggcgcgcctagcagcggCATTTATATTAACTACTTTTCTCCTATGG | (2) |
| 35_CA5377 | gtcagcggccgcatccctgcTAGTTTGTCTTGATACACATATACATATATATATA | (2) |
| 33_CA5377 | GAAAAATGGATGCCAAGTTG | (2) |
| 55_CA6009 | CGGTTTTCACTTAACTGCTGC | (2) |
| 53_CA6009 | cacggcgcgcctagcagcggCATAGCAAGGTGTATGTTTGATTAC | (2) |
| 35_CA6009 | gtcagcggccgcatccctgcTAATTGCATAAACGTGTGATAAATC | (2) |
| 33_CA6009 | TTAGTAGTAGGCGAATAGACACAGAC | (2) |
| 55_CA6323 | TGTTATGTATGAATGCACCGAG | (2) |
| 53_CA6323 | cacggcgcgcctagcagcggCATTGACCCCTGGCTCCG | (2) |
| 35_CA6323 | gtcagcggccgcatccctgcTGACCAATGTACACGACCTATG | (2) |
| 33_CA6323 | TGAATCTTCAATTCATCGGC | (2) |
| 55_CA7221 | ATTGTACGAAGAAGCGGAGC | (2) |
| 53_CA7221 | cacggcgcgcctagcagcggCATTAAAATAAACACTTATAAAGACTACTATC | (2) |
| 35_CA7221 | gtcagcggccgcatccctgcTAGTTTTTTGTTTAGAGTTTGTATATTG | (2) |
| 33_CA7221 | CAAAAGGACAATCAATTGGATG | (2) |
| 55_CA7387 | AAGTCTGACATTACACCAATATAAGG | (2) |
| 53_CA7387 | cacggcgcgcctagcagcggCATTATGTAGTGTGTTTATTAGGAGAAG | (2) |
| 35_CA7387 | gtcagcggccgcatccctgcTAAGACTTGATACTTACAGATTTGTATATG | (2) |
| 33_CA7387 | CTATTGTTGATGGTGAACCACC | (2) |
| 55_CA7402 | CGCTCCTAATTTCTCCAAGC | (2) |
| 53_CA7402 | cacggcgcgcctagcagcggCATTATTAGTATTTGTCAAGTGCACAC | (2) |
| 35_CA7402 | gtcagcggccgcatccctgcTAAAATGTTTTATAGCTAAGCTGTACC | (2) |
| 33_CA7402 | AAATATGGCTTATCGACGGAG | (2) |
| Epitope-tagging cassettes based on fusion PCR strategy | | |
| Name | Sequence (5' - 3') | Reference |
| CA2834_55tag | GCGAGAACATGGGCATTTGA | This study |
| CA2834_53tag | cactagcagcagaaccggaCACATTATTATTTAATTTATCTAATTCAGC | This study |
| CA2834_35tag | attaatttcgataagccaggttaacctgcAGATCAAGTTGGCTGATTGA | This study |
| CA2834_33tag | TTGAAATTTGACGAGGAATT | This study |
| CA2834_9myc_fwd | CTGAATTAGATAAATTAAATAATAATGTGtccggttctgctgctagtgg | This study |
| pFA-backb_rev_CA2834 | TCCCATTATCAATCAGCCAACTTGATCTgcaggttaacctggcttatcg | This study |
| CA6801_55tag | CCTGAAGATTTGGGCGATGT | This study |
| CA6801_53tag | tttgttcaccactagcagcagaaccggaTGAACTTTTATTTAGTTCTTC | This study |
| CA6801_35tag | attaatttcgataagccaggttaacctgcTCATGTAAAGTGTGAGGGAC | This study |
| CA6801_33tag | TTAGTGAGCAAACTGAACGA | This study |
| CA6801_9myc_fwd | AAGAAATTGAAGAACTAAATAAAAGTTCAtccggttctgctgctagtgg | This study |
| pFA-backb_rev_CA6801 | AAATAGTAGTCCCTCACACTTTACATGAgcaggttaacctggcttatcg | This study |
| icCA2834_55HAtag | ttctttcctgcgttatcccctgattctgtggataaccgtaGCGAGAACATGGGCATTTGA | This study |
| CA2834_53HAtag | cgtatgggtaaaagatgttCACATTATTATTTAATTTATCTAATTCAGC | This study |
| CA2834_35HAtag | attaatttcgataagccaggttaacctgcAGATCAAGTTGGCTGATTGA | This study |
| icCA2834_33HAtag | aacgcagaaaatgaaccggggatgcgacgtgcaagattacTTGAAATTTGACGAGGAATT | This study |
| CA2834_3HA_fwd | GAATTAGATAAATTAAATAATAATGTGaacatcttttacccatacgatg | This study |
| pFA-backb_rev_CA2834 | TCCCATTATCAATCAGCCAACTTGATCTgcaggttaacctggcttatcg | This study |
| icCA6801_55HAtag | ttctttcctgcgttatcccctgattctgtggataaccgtaCCTGAAGATTTGGGCGATGT | This study |
| CA6801_53HAtag | caggaacatcgtatgggtaaaagatgttTGAACTTTTATTTAGTTCTTC | This study |
| CA6801_35HAtag | attaatttcgataagccaggttaacctgcTCATGTAAAGTGTGAGGGAC | This study |
| icCA6801_33HAtag | aacgcagaaaatgaaccggggatgcgacgtgcaagattacTTAGTGAGCAAACTGAACGA | This study |
| CA6801_3HA_fwd | GAAATTGAAGAACTAAATAAAAGTTCAaacatcttttacccatacgatg | This study |
| pFA-backb_rev_CA6801 | AAATAGTAGTCCCTCACACTTTACATGAgcaggttaacctggcttatcg | This study |
| CA6506_55tag | TGGGGTGAAGACATGGAAGC | This study |
| CA6506_53tag | gttcaccactagcagcagaaccggaTGACAGAAGAAACTCTAATAATGC | This study |
| CA6506_35tag | aatttcgataagccaggttaacctgcGACCAACGAAACAAAACAAAACA | This study |
| CA6506_33tag | TGGCCAACGACCAATTACTT | This study |
| CA6506_9myc_fwd | AACAAGCATTATTAGAGTTTCTTCTGTCAtccggttctgctgctagtgg | This study |
| pFA-backb_rev_CA6506 | TTTTTTGTTTTGTTTTGTTTCGTTGGTCgcaggttaacctggcttatcg | This study |
| partialRPD31_55tag | ATGGTAGTTGGTGGAGGTGG | This study |
| partialRPD31C_53HAtag | tcaggaacatcgtatgggtaaaagatgttGGAGCCTCCCTTTGTGTCAA | This study |
| partialRPD31C_3HA_fwd | ATGGCAATTGACACAAAGGGAGGCTCCaacatcttttacccatacgatg | This study |
| alpha2_55tag | TGAATTCACATCTGGAGGCAC | This study |
| alpha2_53tag | ttgttcaccactagcagcagaaccggaACCTGTTAATAGCAAAGCAGCC | This study |
| alpha2_35tag | taatttcgataagccaggttaacctgcTCTTCGTATAGGTGTGCACTTT | This study |
| alpha2_33tag | GGTCATGCCTTCCTTATTTGCT | This study |
| alpha2_9myc_fwd | CTGAGTTGGCTGCTTTGCTATTAACAGGTtccggttctgctgctagtgg | This study |
| pFA-backb_rev_alpha2 | AAAATTAAAGTGCACACCTATACGAAGAgcaggttaacctggcttatcg | This study |
| Gene reintegregation cassettes based on fusion PCR strategy | | |
| Name | Sequence (5' - 3') | Reference |
| 3P_SAT_M5 | ccgctgctaggcgcgccgtgAAATGTCGAGCGTCAAAACTAGAG | (2) |
| 3P_SAT_M3 | gcagggatgcggccgctgacCTAGTGGATCTGAAGTTCCTATTCTC | (2) |
| comRPD3_53 | cacggcgcgcctagcagcggTCCCATTATCAATCAGCCAACT | This study |
| comRPD3_35 | gtcagcggccgcatccctgcCTAAGTAGGGGTTTGAAAATAAG | This study |
| comCA6801_53 | cacggcgcgcctagcagcggGTAGTCCCTCACACTTTACATGA | This study |
| comCA6801_35_2 | gtcagcggccgcatccctgcGGATATTTAGATTGTTTGTG | This study |
| partialCA6801_53 | cacggcgcgcctagcagcggTTATTCTATATTATCACCGGTAACG | This study |
| partialCA6801_53_2 | cacggcgcgcctagcagcggTTAGGAGCCTCCCTTTGTGTCAA | This study |
| Quantitative PCR | | |
| Name | Sequence (5' - 3') | Reference |
| RTpWOR1F1 | AGATGACGGCTATACTTGCTCA | This study |
| RTpWOR1R1 | TCTTTCACTCATGCACGCAC | This study |
| RTpWOR1F2 | GACTTTGTACACAGGCTGCA | This study |
| RTpWOR1R2 | CTGTGTTCCTTGTTTCCCACA | This study |
| RT5_CHIP1F | AGCGTAAATTTGGTCCCGAC | This study |
| RT3_CHIP1R | GCAAGCAACATTGGACCTGA | This study |
| RT5_CHIP2F | ACTTTGCTGTTACTGCTACCAC | This study |
| RT3_CHIP2R | GTTGGTCGTTGGTTGTTGGT | This study |
| RT5_CHIP3F | TTTCCTGCTGCACAACACAT | This study |
| RT3_CHIP3R | TCACTCTTCACTCTTCTCCCA | This study |
| RT5_CHIP4F | TCCTTTGCGCTTTTCATTGTT | This study |
| RT3_CHIP4R | ACGGGAAACAGAAAACAGCA | This study |
| RT5_tC_inter | GTGCTATTTACATTCGGTCTTGTTG | This study |
| RT3_tC_inter | TGGGGATGCACAAGATGTAAGTG | This study |
| RT5_CA4884 | GCCAACAACAACAGTAGCTCACG | (2) |
| RT3_CA4884 | TCCATGTGAATTACCACCACCTG | (2) |
| RT5_PAT1 | TTATCGGAATGGTCCTCGTG | (2) |
| RT3_PAT1 | CCAGAAGAACCA TCA TCAAC | (2) |

1. **Noble SM, Johnson AD.** 2005. Strains and Strategies for Large-Scale Gene Deletion Studies of the Diploid Human Fungal Pathogen *Candida albicans*. Eukaryotic Cell **4:**298-309.

2. **Hnisz D, Schwarzmuller T, Kuchler K.** 2009. Transcriptional loops meet chromatin: a dual-layer network controls white-opaque switching in *Candida albicans*. Mol Microbiol **74:**1-15.
