# Supplementary material for: The Paralogous Histone Deacetylases Rpd3 and Rpd31 Play Opposing Roles in Regulating the White-Opaque Switch in the Fungal Pathogen Candida albicans
Source: mBio. 2016 Nov 15;7(6):e01807-16. doi: 10.1128/mBio.01807-16 (PMC5111407; doi:10.1128/mBio.01807-16)
Supplement: Table S4 — Comparison of orthologues involved in Rpd3L and Rpd3S complexes in S. cerevisiae and C. albicans [file mbo006163061st4.docx]

Table S4. Comparison of orthologues involved in Rpd3L and Rpd3S complexes in *S. cerevisiae* and *C. albicans*.

| *S. cerevisiae* | | *C. albicans* | | Protein simililarity (%) |
| --- | --- | --- | --- | --- |
| Protein name | M.W. (KDa) | Orthologous gene | M.W. (KDa) |  |
| Sin3 | 175 | orf19.6011 | 160 | 62 |
| Ume1 | 51 | - | - | - |
| Pho23 | 37 | orf19.1759 | 52 | 49 |
| Rxt2 | 49 | orf19.4615 | 37 | - |
| Rxt3 | 34 | orf19.3568 | 59 | 46 |
| Sds3 | 37 | orf19.1856 | 38 | 49 |
| Dep1 | 47 | orf19.808 | 68 | 51 |
| Sap30 | 23 | orf19.6667 | 19 | 58 |
| Cti6 | 57 | orf19.5617 | 55 | 58 |
| Rco1 | 79 | orf19.6506 | 77 | 42 |
| Eaf3 | 45 | orf19.2660 | 43 | 50 |
| Rpd3 | 48 | orf19.2834 | 54 | 85 |
|  |  | orf19.6801 | 65 | 92 |

- Data not avalible
