# Supplementary material for: The Paralogous Histone Deacetylases Rpd3 and Rpd31 Play Opposing Roles in Regulating the White-Opaque Switch in the Fungal Pathogen Candida albicans
Source: mBio. 2016 Nov 15;7(6):e01807-16. doi: 10.1128/mBio.01807-16 (PMC5111407; doi:10.1128/mBio.01807-16)
Supplement: Table S5 — Epitope tagging does not change the W/O switching of parent strains. [file mbo006163061st5.docx]

Table S5. Epitope tagging didn't change the white to opaque switching of the parent strains.

| Strain | White to opaque switching frequency (%) |
| --- | --- |
| WT | 35.9 ± 2.3 |
| *rpd3*Δ/Δ | 6.4 ± 0.4 ** |
| *rpd31*Δ/Δ | 87.8 ± 2.9 ** |
| *rpd3*Δ/Δ *rpd31*Δ/Δ | 60.5 ± 2.0 * |
| *rpd3*Δ/Δ::*RPD3*-9myc | 26.5 ± 3.6 |
| *rpd31*Δ/Δ::*RPD31*-9myc | 34.8 ± 6.3 |
| Rpd3-3HA Rco1-9myc | 33.7 ± 1.9 |
| Rpd31-3HA Rco1-9myc | 38.5 ± 5.1 |
| WT + α2-myc | 41.7 ± 4.7 |
| *rpd3*Δ/Δ + α2-myc | 4.8 ± 3.1 ** |
| *rpd31*Δ/Δ + α2-myc | 90.2 ± 3.8 ** |
| *rpd3*Δ/Δ *rpd31*Δ/Δ + α2-myc | 65.3 ± 1.2 ** |

Strains were grown on YPD plates at 30 °C for 2 days. Then white cells were plated on Lee’s-GlcNAc medium, 25 °C, 5% CO_2_ for 5 days. White to opaque switching frequency (%) = (opaque + opaque-sectored colonies)/total colonies × 100. * P < 0.05, ** P < 0.01 compared to WT strain.
